# Safe and Sustainable by Design MOF Beads for Selective Entrapment and Recovery of Rare Earth Elements

**DOI:** 10.1021/acs.est.5c03112

**Published:** 2025-08-01

**Authors:** Prathmesh Bhadane, Pankti Dhumal, Emilie Brun, Andrew Britton, Iseult Lynch, Swaroop Chakraborty

**Affiliations:** † School of Geography, Earth & Environmental Sciences, 1724 University of Birmingham, Edgbaston B15 2TT, U.K.; ‡ Materials Engineering, 242275 Indian Institute of Technology, Gandhinagar 382355, India; § School of Chemistry, 4468 University of Leeds, Leeds LS2 9JT, U.K.

**Keywords:** Safe and Sustainable by Design, Metal Organic Framework, Circular Economy, Sustainable Resource Recovery, Green Synthesis, Rare Earth Elements

## Abstract

We report the development of CA-BNMG-1 composite beads-cellulose acetate macrobeads embedded with nanosized copper imidazolate MOFs (BNMG-1) -engineered via nonsolvent-induced phase separation for the selective recovery of rare earth elements (REEs) from complex aqueous environments. This encapsulation strategy ensures uniform MOF dispersion, enhanced mechanical integrity, and minimized Cu­(II) leaching (<1%), fulfilling the Safe and Sustainable by Design (SSbD) criteria. The CA matrix not only mitigates copper toxicity but also enables facile bead handling, recyclability, and scalable deployment in fixed-bed systems. Adsorption studies across a 10-REE standard solution and two simulated waste streams demonstrated significantly improved REE selectivity over pristine BNMG-1. Separation factors (SFs) for Yb­(III) over Mn­(II), Ni­(II), and Na­(I) reached 194.5, 325.8, and 339, respectively; Eu­(III) showed SFs of 155.5, 260.5, and 271.2. The beads retained over 95% of their uptake capacity across multiple adsorption and single desorption cycles using mild acidic eluents, confirming excellent reusability and structural stability. This work advances a robust, low-toxicity, and scalable REE recovery platform that integrates adsorptive performance with environmental safety. CA-BNMG-1 beads offer a compelling alternative to solvent extraction, with potential for integration into circular economy strategies targeting REE recovery from e-waste, mine tailings, and industrial effluents-addressing both resource security and sustainability challenges.

## Graphical abstract

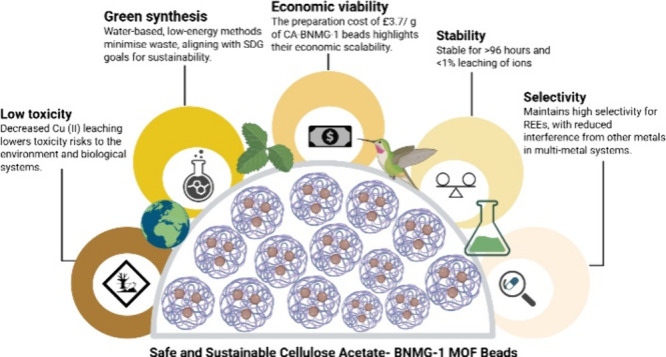

## 1 Introduction

Rare Earth Elements (REEs) are indispensable to modern technology, underpinning industries such as renewable energy, electronics, defense, and advanced materials. Their unique properties, including magnetism, luminescence, and catalysis, make them critical components in applications ranging from wind turbines and electric vehicles to medical imaging and advanced optics.^1,2^ Despite their name, REEs are relatively abundant in the Earth’s crust, but their dispersed nature and low concentrations present significant challenges for their extraction and recovery.^3^ This dispersion necessitates intensive and environmentally costly mining processes, creating a pressing need for more sustainable and efficient recovery methods to increase the circularity of REEs. The increasing global demand for REEs has driven research into alternative solutions, particularly focusing on recovering these valuable elements from secondary sources such as e-waste, coal ash, waste rock, and acid mine drainage.^4,5^ Such approaches address both the growing demand for REEs and the need for sustainable management of industrial waste streams. In addition to this waste, the ore purification process, mining and smelting of REEs generate waste streams containing 30 to 40 mg/L REEs concentrations in combination with other metal ions.^6^

Conventional recovery methods, including pyrometallurgy, electrometallurgy and hydrometallurgy, are widely used but pose serious environmental concerns.^7–9^ These techniques are associated with high energy consumption, the emission of toxic gases, and the generation of hazardous residues due to the extensive use of chemical reagents. In contrast, adsorption technologies have emerged as a promising alternative, offering the potential for high efficiency, selectivity, and reusability. Among the advanced materials explored for adsorption, Metal–Organic Frameworks (MOFs) stand out due to their high surface area, tunable pore structures, and exceptional capability to selectively adsorb metal ions.^10–12^ MOFs have shown promise in addressing the challenges of REE recovery; however, their practical application remains constrained by several critical issues.^13^ One major limitation of conventional MOFs lies in their instability in aqueous environments, where weak metal–ligand bonds can lead to hydrolysis and the subsequent leaching of metal ions. This not only diminishes their adsorption capacity but also raises significant environmental and safety concerns.^14^ Further, the fine powder form of most MOFs complicates their recovery from solutions or from the environment postadsorption, requiring labor-intensive and costly separation processes. Additionally, the complex synthesis methods and limited recyclability of MOFs make them economically and practically challenging for large-scale applications.^15,16^

## 2 CA-BNMG-1 Beads as a Scalable, Safe, and Sustainable System

This study introduces a Safe and Sustainable by Design (SSbD)^17–20^ approach that integrates BNMG-1 MOFs within a biobased cellulose acetate (CA) polymer matrix to develop CA-MOF composite beads for REE recovery. Unlike traditional MOF-based adsorbents, which suffer from hazardous solvent use, poor stability, and operational challenges, our bead-based design addresses these limitations by eliminating highly toxic solvents such as DMF from the MOF synthesis process and using DMSO-a low-toxicity solvent widely employed in pharmaceutical and biological applications—only in the polymer processing step. The use of DMSO was confined to a closed-loop system and followed by thorough washing, ensuring minimal environmental impact.

The BNMG-1 MOFs are hydrolytically stable and synthesized in water, ensuring a greener alternative to conventional MOFs. The CA matrix provides mechanical stability, minimizes environmental contamination, and allows for easy recovery from aqueous solutions, overcoming the separation difficulties associated with fine MOF powders. Conducted under ambient conditions, the synthesis process significantly reduces energy consumption, improving economic viability and aligning with green chemistry principles by replacing hazardous substances and utilizing renewable materials.

The prepared beads demonstrate exceptional adsorption efficiency and selectivity, particularly for Yb and Eu, even in complex multimetal systems. Retaining 95% of their adsorption capacity over five cycles, they exhibit high durability and waste minimization, making them a cost-effective solution for large-scale applications. Tables S4.1 and S4.2 provide a comparative analysis, highlighting their superior sustainability, recyclability, and adsorption performance over conventional REE adsorbents, including silica-, carbon-, polymer-, and hydrometallurgical-based methods. The CA-BNMG-1 beads offer notable economic viability, with competitive production costs and reduced operational complexity, positioning them as a cost-effective alternative to traditional REE adsorbents. Their scalable manufacturing process, detailed through unit economics analysis (Supplementary Table S4.6), demonstrates strong potential for industrial adoption and economic sustainability. By integrating green chemistry principles with innovative material design, this study advances safer, more sustainable MOF applications in environmental remediation and critical metal recovery, setting a new standard for advanced materials in resource management. Our CA-BNMG-1 composite beads have recently been demonstrated to selectively remediate lead (Pb) from complex water matrices, confirming their versatility across different critical-metal recovery applications.^21^ This study presents a scalable and sustainable solution for rare earth element REE recovery using CA-BNMG-1 composite beads, integrating biodegradable cellulose acetate (CA) with BNMG-1 MOFs. Unlike traditional MOF-based adsorbents, which often rely on hazardous organic solvents and exhibit poor stability in aqueous systems, our design significantly reduces toxicity risks, enhances reusability, and aligns with SSbD framework (Table S4.1).^17,18,22^

## 3 Experimental Section

BNMG-1 MOFs were synthesized through an aqueous-based method using copper nitrate and 2-methylimidazole at ambient temperature. The resultant nanosized MOFs were thoroughly washed and vacuum-dried to obtain the activated material. Composite beads were then prepared by integrating 20 wt.% BNMG-1 into a CA matrix using a nonsolvent-induced phase-separation technique. Specifically, CA dissolved in dimethyl sulfoxide (DMSO) containing dispersed BNMG-1 was extruded dropwise into water, resulting in immediate bead formation upon contact. The beads were washed repeatedly to remove residual DMSO, followed by drying at ambient temperature (Figure 1A and 1B).

**Figure 1:**
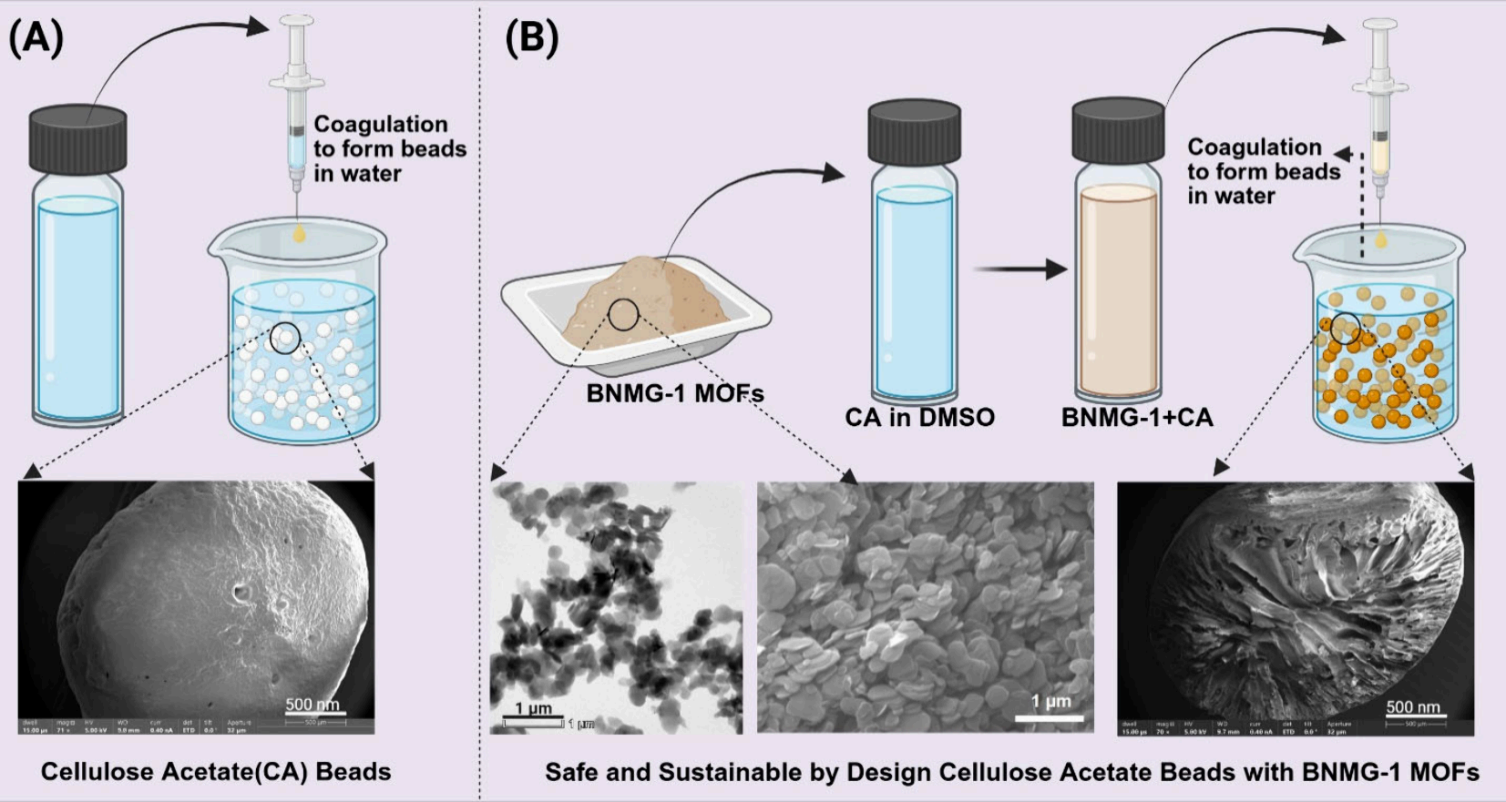
Synthesis and structural characterization of CA and BNMG-1 composite beads: (A) Synthesis process of CA beads, with representative SEM image showing their surface morphology. (B) Synthesis process of CA-BNMG-1 beads, incorporating BNMG-1 MOF into the polymer matrix. The panel includes a TEM and SEM image of the BNMG-1 MOFs showing their sheet-like two-dimensional structure, and a cross-sectional SEM image of the CA-BNMG-1 bead.

Batch adsorption experiments were conducted using solutions containing mixtures of REEs, including Ytterbium (Yb) and Europium (Eu), in various complex matrices. Adsorption efficiencies, kinetics, isotherms, and selectivity assessments were quantified using inductively coupled plasma mass spectrometry (ICP-MS). The structural integrity, surface chemistry, and MOF dispersion in the composite beads were characterized by X-ray diffraction (XRD), Fourier-transform infrared spectroscopy (FTIR), scanning electron microscopy coupled with energy-dispersive spectroscopy (SEM-EDS), and X-ray photoelectron spectroscopy (XPS). Cytotoxicity tests were performed using zebrafish embryonic (ZF4) cells to confirm reduced toxicity and environmental safety of the composite beads. Detailed protocols for synthesis, characterization, adsorption testing, and cytotoxicity assessment are fully described in the Supporting Information (Sections S1.1–S1.5).

## 4 Results and Discussion

### 4.1 Physiochemical Characterization of CA and CA-BNMG-1 Beads

The synthesized BNMG-1 MOF exhibits sheet-like morphology as shown in the TEM and SEM micrographs (Figure 1B) with individual sheets measuring between 10 and 18 nm in thickness and lateral dimensions ranging from 250 to 500 nm. The surface and cross-sectional features micrographs of the CA beads (Figure 1A and Figure S3.1A–C) revealed a porous structure of the beads because of the phase separation process. In contrast, the micrographs of the CA-MOF beads (Figure 1B and Figure S3.1D–F, I) displayed a more compact cross-sectional surface morphology because of the presence of BNMG-1 MOFs in CA matrix. The successful incorporation of BNMG-1 within the CA matrix was evidenced by distinct morphological features associated with BNMG-1. The reinforcement of the CA with BNMG-1 nanosheets was further confirmed through energy dispersive X-ray spectroscopy (EDS) mapping (Figure S3.1G,H), which highlighted a uniform distribution of Cu throughout the composite structure. This even distribution indicates effective impregnation of BNMG-1 within the CA matrix, ensuring enhanced interaction sites for adsorption while maintaining the structural integrity of the composite. The combined SEM and EDS analyses demonstrate the successful synthesis of CA-BNMG-1 composite beads with a well-dispersed BNMG-1 phase, crucial for their application in REE adsorption.

The FTIR spectrum of BNMG-1 MOFs (Figure S3.2A and Table S4.3) shows characteristic peaks associated with the 2-Methylimidazole (2-MeIM) linker, including peaks at 1423 cm^–1^ and 1307 cm^–1^ for C–N stretching and a peak at 1150 cm^–1^ for C–H stretching.^26^ Additional strong bands at 1570 cm^–1^ and 1618 cm^–1^ represent −C=N stretching, while the absorption peak at 431 cm^–1^ confirms the formation of the Cu–N bond, validating the coordination between Cu and the 2-MeIM linker in the BNMG-1 particles. The FTIR characterization of pure CA beads and composite beads, shown in Figure S3.2A, provides insights into their structural features and interactions. In the FTIR spectra of CA beads, characteristic peaks appear at 1737 cm^–1^, 1440 cm^–1^, and 1215 cm^–1^, corresponding to C=O stretching, H–C–H vibrations, and C–O stretching, respectively.^27^ These bands highlight the ester linkages and functional groups within CA, which enable potential interactions when forming composites. In the CA-BNMG-1 composite beads, the FTIR spectrum reveals that the characteristic peaks of BNMG-1 (−Cu–N– around 440 cm^–1^ and −C=N at 1618 cm^–1^) are largely retained. Some characteristic peaks, like the 1307 cm^–1^ (C–N stretching of 2-MeIM), are not clearly observed, likely due to overlap with CA bands. Meanwhile, the intensified peaks at 1737 cm^–1^ and 1618 cm^–1^, corresponding to C=O and C=N stretching respectively, may indicate strengthened interactions or bonding within the composite. This absence of specific peaks may result from overlap of BNMG-1 peaks with CA’s bands because of the dilution effect, while the increase in peak intensity suggests enhanced interaction within the composite. This enhancement is not merely additive but suggests structural interactions between CA and BNMG-1, such as hydrogen bonding between CA’s carbonyl groups and the MOF’s imidazole N–H or surface hydroxyls. Such interactions can stabilize the composite by altering electron density distributions, intensifying specific vibrational modes. A broad peak near 3650 cm^–1^, assigned to O–H or N–H stretching, further supports hydrogen-bonding networks at the CA-MOF interface. This behavior aligns with findings in other MOF-CA systems, such as PZIF-8@CA, where the incorporation of ZIF-8 MOFs strengthened CA’s characteristic bond intensities, implying cross-linking or hydrogen bonding between CA’s carbonyl groups and the MOF.^28,29^ BNMG-1’s structural integration in CA matrix is further supported by the retention of Cu–N coordination bond, confirming that the MOF framework remains intact and stable within the composite. Ester group interaction is reflected in the subtle shift of the C=O peak from 1741 cm^–1^ to 1749 cm^–1^, indicating partial electron redistribution due to interfacial bonding between CA and BNMG-1. These observations collectively suggest that the composite is physically blended yet interactively stabilized, with hydrogen bonding and interfacial interactions playing a key role in its structural synergy.

XRD analysis (Figure S3.2B) of BNMG-1 displayed its primary characteristic peaks at 14.7°, 29.8°, 33.2°, and 47.7°.^23,30^ However, complete structural resolution was hindered by the small crystal size of BNMG-1. These distinctive peaks were prominently retained in the CA-BNMG-1 composite, alongside a hump around 22.5° corresponding to the disordered structure of CA.^30^ This observation confirms the structural integrity of BNMG-1, even after incorporation into the CA matrix. The thermogravimetric analysis (TGA) and differential thermogravimetry (DTG) curves presented in Figure S3.2C, D illustrates that the reinforcement of BNMG-1 in the CA beads impacted their thermal stability, with CA-BNMG-1 beads showing a slightly earlier onset of degradation compared to the pure CA beads. This behavior likely results from synergistic interactions between the CA and MOF components within the composite. BNMG-1 exhibited a three-stage degradation pattern, which was similarly observed in the CA-BNMG-1 beads. The initial mass loss stage is attributed to the release of moisture or water content. The BNMG-1 remained stable between 30 and 240 °C. Following this, there was a sharp mass loss of approximately 25% around 250 °C, likely due to the degradation of the free 2-MeIM ligand. Between 250 and 500 °C, a gradual weight loss of around 50% was observed, corresponding to the complete decomposition of the 2-MeIM linker and the formation of final residues. This three-stage degradation pattern was also evident in the CA-BNMG-1 beads, as indicated by the DTG curve, which showed a small mass loss around 220 °C. This early degradation is attributed to the release of unreacted 2-MeIM from BNMG-1. A significant mass loss (∼70%) was observed between 340 and 350 °C, consistent with the thermal degradation of CA. Each CA-BNMG-1 bead weighs between 2.5 and 3 mg, with each bead containing approximately 0.72 mg of BNMG-1. This was determined from the Cu­(II) content, which is about 0.16 mg per bead, as measured by ICP-MS. Given that BNMG-1 comprises approximately 22.4% Cu­(II), the MOF accounts for about 20–30% of the CA-BNMG-1 bead. This is consistent with the initial addition of 20 wt % BNMG-1 to the CA solution during bead synthesis. The slightly higher calculated percentage of BNMG-1 may be attributed to the loss of CA during the handling and preparation stages, including some adherence to the syringe walls.

### 4.2 REE Adsorption Enhanced with CA-BNMG-1 Composite Beads

In the preliminary adsorption studies (Figure 2A), the affinity of BNMG-1 MOF, CA, and CA-MOF composite beads toward various REEs was evaluated. The results revealed that the BNMG-1 MOF exhibited a nearly uniform affinity across all REEs, primarily due to the high number of available binding sites, except for lanthanum (La). As shown in Figure 2B, the separation factor (SF) of BNMG-1 for a representative REE-1 relative to another REE-2 ranged from 1 to 2, indicating a narrow SF range (eq S3 and S4). However, a significant limitation was observed when using standalone BNMG-1. Approximately 20% of Cu­(II) ions leached into the aqueous solution over the 24 h adsorption cycle, highlighting the material’s instability in water and raising concerns about potential toxicity due to copper release. Furthermore, the fine powder form of BNMG-1 complicates recovery of REE adsorbed BNMG-1 from solution post-adsorption, limiting its practical utility in aqueous systems, as demonstrated in Figure 3A.

**Figure 2:**
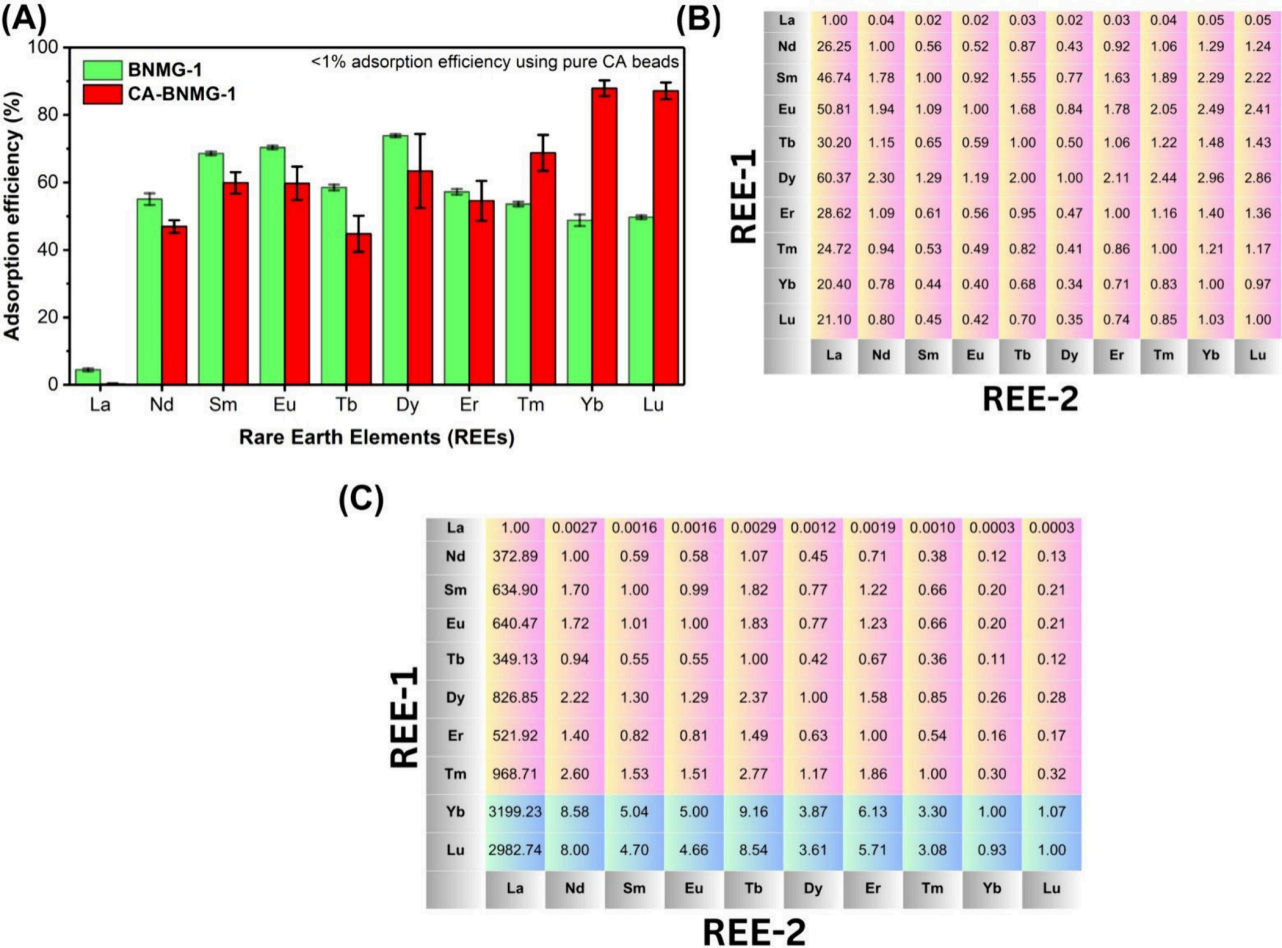
Comparative adsorption performance of BNMG-1 MOF and CA-BNMG-1 composite beads: (A) Adsorption efficiency of heavy and light REEs from a 20 mg/L solution (each REE contained in a single mixture) using BNMG-1 MOF (1000 mg/L) and CA-BNMG-1 beads (5 beads/mL). (B, C) Separation factors (SF) for REE-1 versus REE-2 using BNMG-1 MOF and CA-BNMG-1 composite beads, respectively, demonstrating the effectiveness of the composite beads for better selectivity and REE recovery. All adsorption studies were performed in triplicate (*N* = 3) to ensure the reproducibility and reliability of the results.

**Figure 3:**
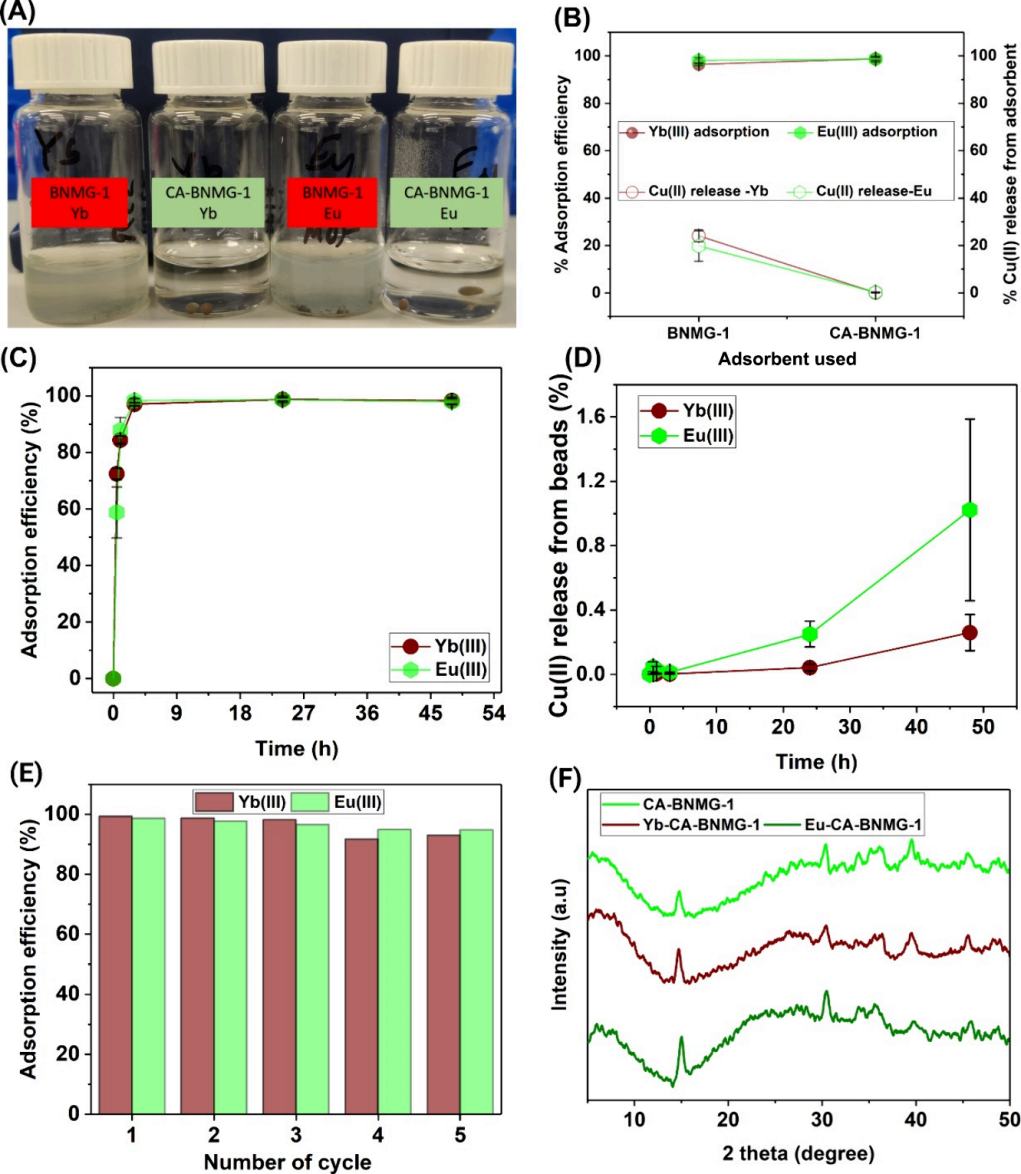
Comprehensive analysis of Yb and Eu adsorption onto MOF composite beads: (A) Visual representation of BNMG-1 MOF and CA-MOF beads suspended in water. (B) Comparative release of copper ions from standalone MOF and composite beads after 24 h in 10 mg/L Yb and Eu solutions. (C) Adsorption kinetics of Yb and Eu onto CA-BNMG-1 beads over various time intervals, demonstrating adsorption rates and equilibrium behavior. (D) Time-dependent release of Cu­(II) from CA-BNMG-1 beads during adsorption. (E) Reusability performance of CA-BNMG-1 beads for Yb and Eu adsorption over multiple cycles using 10 mg/L solutions of Yb and Eu. (F) Post-adsorption XRD analysis of composite beads after Yb or Eu adsorption.

In contrast, the CA-BNMG-1 composite beads showed a more distinctive selectivity pattern. These beads exhibited higher SF values (Figure 2C) for heavy REEs, particularly lutetium (Lu), ytterbium (Yb), and dysprosium (Dy), compared to lighter REEs. For instance, the SF of the CA-BNMG-1 for Lu relative to Sm, Nd, and Eu was 4.75, 8.00, and 4.66, respectively, while for Yb relative to Nd, Sm, and Eu the SF was 8.58, 5.04, and 5.00, respectively. Additionally, Lu and Yb demonstrated higher affinities for CA-BNMG-1 beads than other heavy REEs, such as Dy, Tb, Tm, and Er, as shown in Figure 2A,C. This enhanced adsorption of Lu and Yb by CA-BNMG-1 beads can be attributed to the lanthanide contraction effect, wherein the ionic radii of lanthanides decreased with an increase in atomic number due to imperfect electron shielding.^31^ Consequently, heavy REEs, which possess smaller ionic radii, exhibit stronger interactions with the CA-BNMG-1 matrix compared to lighter REEs, such as La. This effect is particularly evident in multicomponent systems, which tend to Favor heavy REEs over light REEs like La.^32^ We hypothesize that in the composite, the encapsulation of BNMG-1 within the CA matrix restricts the availability of adsorption sites compared to bare BNMG-1. This limited accessibility likely drives preferential adsorption of heavy REEs over light REEs, as heavy REEs exhibit stronger interactions due to their smaller ionic radii and higher charge density. A slight increase in REE concentrations was detected after contact with the blank CA beads (with less than 1% REE adsorption). This increase is not due to adsorption or desorption but likely results from the slight water adsorption in porous CA bead. Upon immersion, the CA beads can absorb water, effectively reducing the free solution volume. This reduction leads to a slight apparent increase in measured REE concentrations, rather than reflecting any actual interaction with the adsorbent. These results confirm that the CA matrix itself does not contribute to REE adsorption, and that the observed selectivity and uptake are attributable to the MOF component in the composite beads.

### 4.3 Adsorption Kinetics, Isotherm and Selectivity Study

To understand the adsorption kinetics and isotherm behavior of Yb­(III) and Eu­(III) onto CA-BNMG-1 beads, time- and concentration-dependent adsorption studies were conducted. As shown in Figure 3C, over 90% of Yb­(III) and Eu­(III) were adsorbed onto the composite beads within 3 h, with adsorption equilibrium achieved at 24 h, reaching 99% adsorption efficiency calculated using eq S1. Compared to the standalone BNMG-1 MOF, the CA-BNMG-1 beads demonstrated significantly enhanced stability during the adsorption process, with less than 1% Cu­(II) release observed after 24 h (Figure 3B,D). This marked reduction in copper ion leaching highlights the effectiveness of the CA matrix in encapsulating the MOF, thereby preventing its direct exposure to the aqueous environment. This shielding effect not only minimizes the potential toxicity associated with Cu­(II) release but also ensures the structural integrity of BNMG-1. Furthermore, despite the reduced leaching, the CA-BNMG-1 beads maintained high adsorption performance, showcasing their ability to balance environmental safety and functional efficiency, making them a promising candidate for sustainable and scalable REE recovery applications. The adsorption kinetics were further analyzed using pseudo-first order (eq S5) and pseudo-second-order (eq S6) model fittings (Figure S3.3). The results, summarized in Table S4.4, indicate that the pseudo-second-order model provided a superior fit for both Yb­(III) and Eu­(III) adsorption, with higher correlation coefficients and equilibrium adsorption capacities than the pseudo-first-order model. These data show that the adsorption of Yb­(III) and Eu­(III) is mainly driven by the chemical interactions between REE, and the functional groups present on the CA-BNMG-1 beads.

REEs can interact with CA-BNMG-1 beads through two primary mechanisms involving coordination with the functional groups present in the BNMG-1 framework. The first mechanism involves the interaction of REEs with oxygen-containing functional groups, leading to the formation of -O-REE bonds. The second mechanism occurs through coordination with nitrogen-containing functional groups, resulting in -N-REE bonds. These interactions are facilitated by the high affinity of REEs for electron-rich donor atoms such as oxygen and nitrogen, which are abundant in the CA-BNMG-1 bead structure. Similar bonding behavior has been reported by Cho et al. with ZIF-8 MOFs,^33^ where REEs were observed to form Zn–O-REE and Zn–N-REE bonds.

An isotherm adsorption study was conducted to elucidate the interaction between Yb­(III), Eu­(III), and CA-BNMG-1 composite beads. The results indicated that as the concentrations of Yb­(III) and Eu­(III) were incrementally increased, the adsorption efficiency correspondingly improved. To confirm that equilibrium is achieved at high concentrations, additional adsorption kinetics experiments were performed using 1000 mg/L solutions of Yb­(III) and Eu­(III) with 5 beads/mL. As shown in Figure S3.4, both Yb­(III) and Eu­(III) exhibited rapid adsorption within the first 6 h, followed by a plateau in adsorption capacity. For Yb­(III), the adsorption capacity reached approximately 49 mg/g at 24 h and showed only a minor increase to about 51 mg/g at 48 h. Similarly, Eu­(III) reached an adsorption capacity of around 22 mg/g at 24 h, with negligible change observed at 48 h. The minimal difference in adsorption capacity between 24 and 48 h, within experimental error, confirms that equilibrium was attained within 24 h for both ions at this high concentration. Therefore, the 24-h contact time used in the isotherm studies is sufficient to ensure equilibrium across the tested concentration range. The equilibrium adsorption capacities (Qe) reached values of 83 μg/bead for Yb­(III) and 55 μg/bead for Eu­(III), with minimal Cu­(II) leaching, even at a high REE solution concentration of 1000 mg/L (Figure S3.5A,B, eq S2). Recalculating the adsorption in terms of the amount of BNMG-1 used (i.e., the concentration of MOFs in the bead) the adsorption capacity based on Langmuir model (Table S4.4) for Yb­(III) and Eu­(III) are 147.5 and 86.4 mg/g of BNMG-1 respectively. These calculations were done considering 0.72 mg of BNMG-1 present per CA-BNMG-1 bead. Although such elevated REE concentrations are not reflective of typical real-life REE leachate solutions, they were applied here to provide a more comprehensive understanding of the adsorption mechanism under controlled experimental conditions.

To thoroughly evaluate adsorption mechanisms, the data were analyzed using both linear and nonlinear forms (Figure S3.6) of Langmuir (eq S7) and Freundlich (eq S8) isotherm models.^34,35^ Initial linear regression suggested superior correlation with the Langmuir model, yielding maximum adsorption capacities of 42.4 mg/g CA-BNMG-1 for Yb­(III) and 24.8 mg/g CA-BNMG-1 for Eu­(III) (Table S4.4). However, nonlinear regression (Figure S3.6A,B), recommended addressing error structure limitations of linearization, provided critical refinements. For Yb­(III), both LangmuirEXT1 (nonlinear *R* ^2^ = 0.928, adsorption capacity = 49.9 mg/g) and Freundlich (*R* ^2^ = 0.924, K_f_ = 0.50) models showed comparable fits, with Langmuir’s theoretical monolayer capacity aligning better with experimental saturation trends.^36,37^ In contrast, Eu­(III) adsorption was best described by the Freundlich model (nonlinear *R* ^2^ = 0.800, K_f_ = 5.09), as the LangmuirEXT1 model produced unrealistic parameters, confirming heterogeneous adsorption. The Freundlich exponent (1/*n* = 0.20 for Eu­(III), 0.62 for Yb­(III)) indicates favorable adsorption at lower concentrations, consistent with the composite’s heterogeneous structure. The adsorption behavior of Yb­(III) and Eu­(III) was effectively described by the Langmuir–Freundlich (Sips) isotherm model (eq S10, Figure S3.6C), capturing both the adsorption capacity and surface heterogeneity of the adsorbent. The fitted parameters revealed higher adsorption capacity and affinity for Yb (*Q*_max_ = 37.15 mg/g, *K* = 0.024) compared to Eu (*Q*_max_= 23.7 mg/g, *K* = 0.010), with both showing moderate site heterogeneity (*n* < 1). High *R* ^2^ values (>0.9) confirmed strong agreement with experimental data, validating the model’s suitability for Yb­(III) and Eu­(III) ion adsorption. Cellulose acetate provides a disordered network of oxygen-rich functional groups (e.g., acetyl, hydroxyl), while the BNMG-1 MOF contributes ordered nanopores and nitrogen-containing ligands (imidazole groups). This duality creates energetically heterogeneous adsorption sites with varying affinities for Yb­(III) and Eu­(III), explaining the divergence in model performance. Combined with pseudo-second-order kinetics, these results confirm chemisorption as the dominant mechanism, highlighting the composite beads’ effectiveness for selective rare earth element recovery.

In addition, the separation factor (SF, presented as the dimensionless constant *R*_L_) calculated using eq S9, can be categorized into four types: *R*_L_ = 0 (irreversible), 0 < *R*_L_ < 1 (favorable), *R*_L_ = 1 (linear), and *R*_L_ > 1 (unfavorable).^38^ The *R*_L_ for both metal ions fell between 0 and 1, confirming favorable adsorption conditions for the CA-BNMG-1 beads. Furthermore, under identical adsorption conditions, the higher Qe value observed for Yb­(III) compared to Eu­(III) highlights the superior affinity of CA-BNMG-1 beads for heavy REE over light REE. This also validates results obtained in the mixed REE system shown in Figure 2. The findings from the kinetic and isotherm adsorption studies underscore the substantial affinity of composite beads for Yb­(III) and Eu­(III), with chemisorption and heterogeneous adsorption mechanism.

Unlike powdered MOFs, which necessitate additional separation steps such as centrifugation or nanoscale filtration to recover the MOF powder after REE adsorption, CA-BNMG-1 beads provide a notable advantage in their ease of removal. Due to their millimeter-scale size, these beads can be easily separated from the solution using a simple filtration net or sieve, eliminating the need for complex and energy-dependent separation processes. These properties highlight the enhanced stability and adsorption efficiency of beads for the recovery of Yb­(III) and Eu­(III), making them a promising candidate for REE recovery in sustainable environmental remediation applications. As illustrated in Figure 3E, the CA-BNMG-1 beads exhibit exceptional adsorption performance across multiple cycles, demonstrating their potential for long-term use. While the five-adsorption cycle study provides promising preliminary evidence of reusability, it does not serve as a definitive measure of long-term durability. Future investigations will involve extending the number of adsorption cycles under accelerated conditions, such as shortened contact times and evaluating performance in continuous flow systems like packed-bed columns. These approaches will better simulate operational environments, allowing for assessment of material fatigue, mechanical stress, and real-world regeneration behavior. Thus, the current durability assessment should be viewed as a proof-of-concept demonstration that establishes a foundation for future optimization.

XRD analysis (Figure 3F) further supports this, showing that the characteristic peaks of both CA and BNMG-1 remain intact after exposure to aqueous solutions containing Yb­(III) and Eu­(III). Moreover, long-term stability studies revealed minimal Cu­(II) release, with only 3–5% observed over 5 cycles of 24-h adsorption cycles, confirming the durability and reliability of CA-BNMG-1 beads for extended applications. All adsorbed REEs were desorbed back into aqueous solution with more than 95% efficiency using 1% HNO_3_ solution and after desorption, REEs can be separately precipitated in their hydroxide form using 1:1 NH_4_OH (Section S3.7, Figure S3.7). As an alternative, sodium citrate can be used to desorb adsorbed REEs back into aqueous solution.^39^ Prepared CA-BNMG-1 beads are stable and effective in the pH range of 3–7, which covers most practical scenarios for mine wastewater and e-waste leachate treatment. For complex matrices with pH below 3, such as certain untreated mine wastewaters or e-waste leachates, we recommend adjusting the pH to within the 3–7 range prior to adsorption or performing shorter contact time adsorption to reduce the risk of MOF degradation. This approach balances adsorption efficiency with structural stability, ensuring the beads’ suitability for realistic environmental remediation applications.

Selectivity test results, presented in Figure 4, evaluate the ability of CA-BNMG-1 beads to preferentially adsorb various REEs. The study employed two distinct combinations of complex solutions, designed to simulate real-world conditions based on the typical compositions of mine wastewater, landfill leachates, and E-waste, including sources like NdFeB magnets, fluorescent lamps, and battery waste.^40–44^ To assess the MOF composite beads’ selectivity for REE, coexisting ions such as sodium (Na), calcium (Ca), and iron (Fe) were included alongside heavy metals commonly found in higher concentrations, such as manganese (Mn), nickel (Ni), and cadmium (Cd). In the first set of experiments (Yb, Eu, Mn, Ni, Na), the metal ions were introduced at nearly equal concentrations of approximately 40 mg/L to evaluate the beads’ selectivity under uniform conditions. In the second set of experiments (Nd, Dy, Fe, Ca, Cd), Cd­(II) was added at three times the concentration of the other ions to assess the beads’ performance under conditions where one ions concentration was disproportionately higher. As depicted in Figure 4A, the prepared composite beads demonstrated exceptional affinity for Yb and Eu over coexisting metal ions such as Mn­(II), Ni­(II), and Na­(I). Over 85% of Yb­(III) and Eu­(III) were adsorbed, whereas the adsorption of Mn­(II), Ni­(II), and Na­(I) remained below 5%. From the initial and final concentrations of the metal ions, *K*_d_ and SF values were calculated, as shown in Figure 4B and 4C. These metrics further highlight the superior selectivity of CA-BNMG-1 beads for REEs. Specifically, the SF values for Yb­(III) relative to Mn­(II), Ni­(II), and Na­(I) were 194.5, 325.8, and 339, respectively, indicating that Yb­(III) exhibited nearly 195 times higher affinity for the beads than Mn­(II) and over 300 times higher affinity compared to Ni­(II) and Na­(I). Similarly, SF values for Eu­(III) relative to Mn­(II), Ni­(II), and Na­(I), presented in Figure 4C, reinforce this strong selectivity trend.

**Figure 4:**
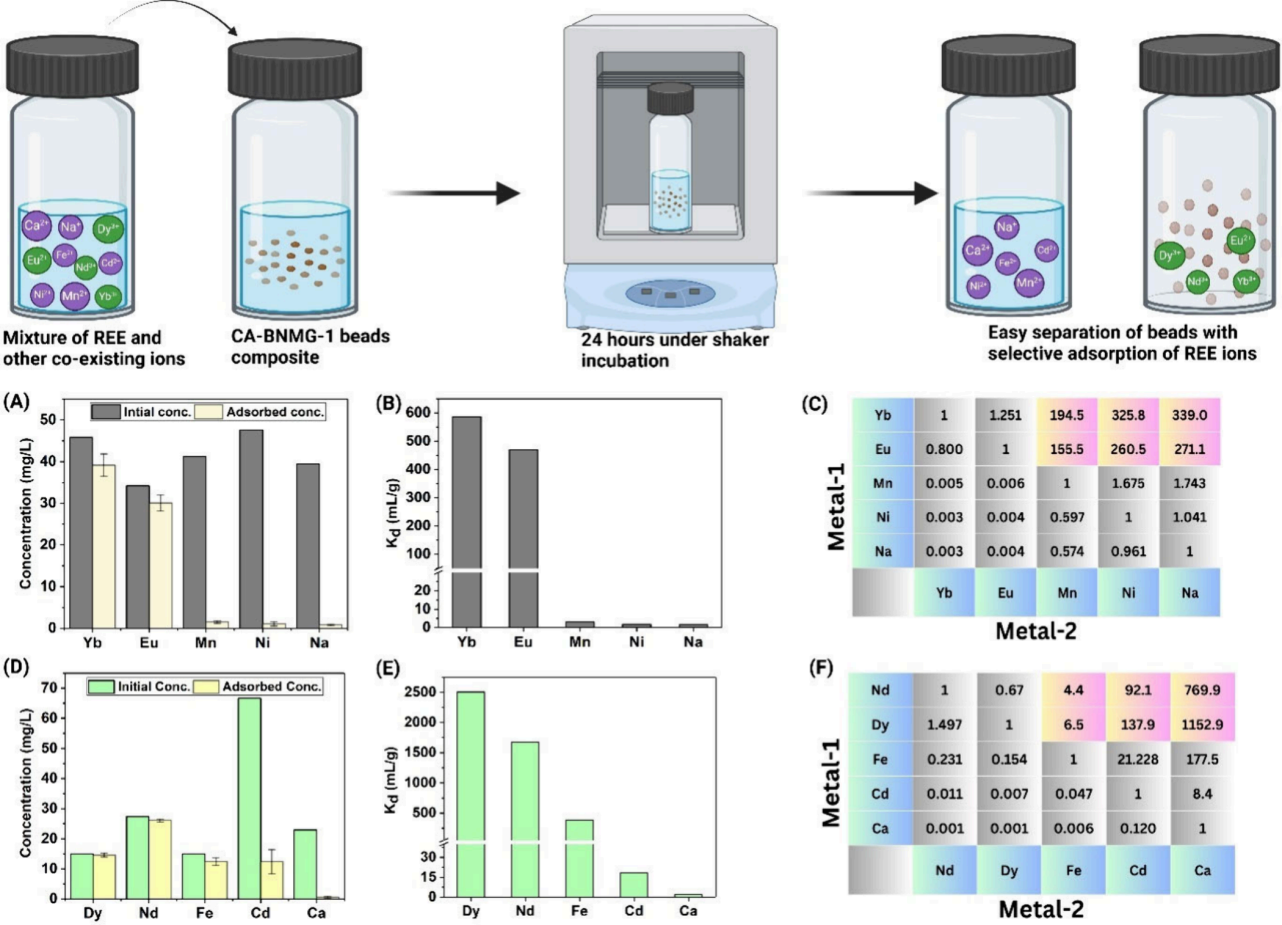
Selectivity assessment of CA-BNMG-1 beads for REEs in simulated mine wastewater and e-waste leachate. Metal ion concentrations were chosen based on reported values in the literature to replicate realistic conditions.^6^ (A–C) For the first set of metals, initial and adsorbed concentrations, distribution coefficients (*K*_d_ values), and separation factors (SF) demonstrate the preferential adsorption of REEs over competing ions. (D–F) For the second set of metals, initial and adsorbed concentrations, *K*_d_ values, and SF confirm the preferential adsorption of REEs by CA-BNMG-1 beads in the presence of competing metal ions.

In the second set of experiments (Figure 4D–F), where Cd­(II) was introduced at a concentration three times higher than other ions, the beads continued to exhibit successful adsorption of REEs. The SF values for Nd­(III) and Dy­(III) relative to Cd­(II) were 92.1 and 137.9, respectively, highlighting the beads’ ability to maintain selective adsorption even in the presence of a dominant ion like Cd­(II). Additionally, no significant interference from Ca­(II) was observed in REE adsorption, with Nd­(III) and Dy­(III) showing SFs of 769.9 and 1152.9 relative to Ca­(II). However, Fe­(III) showed moderate affinity for the beads, likely due to the coordination interaction with carboxylate groups present on the bead surface.^45^ Despite this, Nd­(III) and Dy­(III) still demonstrated SFs of 4.4 and 6.5 relative to Fe­(III), with *K*_d_ values Nd­(III) (1675.6 mL/g) and Dy­(III) (2509.2 mL/g) indicating higher affinity for the beads compared to Fe­(III) (386.3 mL/g). We also compared the adsorption efficiency of Yb­(III) with that of metal ions possessing different valencies; detailed results are provided in section S-3.8 and Figure S3.8. While anion-specific effects were not the focus of this work. From literature it can be seen that there is a minimal effect of anions on the adsorption of REEs (Table S4.5). These findings clearly highlight the effectiveness of CA-BNMG-1 beads as selective and efficient materials for recovering REEs from complex aqueous solutions, including waste streams, where competing ions and varying concentrations are prevalent.

Post-adsorption SEM-EDS (Figure S3.9A, B) analysis unequivocally confirmed the presence of Yb and Eu on the surface of CA-BNMG-1 beads, providing strong evidence of successful adsorption. The FTIR spectra of composite beads exhibited marked differences as a result of REE adsorption. As illustrated in Figure 5A, the characteristic peaks associated with 2-MeIM exhibited significant attenuation following Yb­(III) and Eu­(III) adsorption. Notably, the in-plane bending vibration of the imidazole ring around 1330 cm^–1^, the ring vibration modes in the 900–1500 cm^–1^ range, and the C–H bending peak near 770 cm^–1^ were markedly diminished, indicating disruption of the imidazole framework due to REE interaction. Concurrently, the appearance of new peaks near 1580 cm^–1^ and within the 400–500 cm^–1^ region suggests the formation of coordination bonds between REE­(III) ions and nitrogen (−N)/oxygen (−O) donor groups present on the CA-BNMG-1 surface. XPS analysis confirmed the successful adsorption of REEs onto CA-BNMG-1 beads, with Eu and Yb showing surface atomic percentages of 13.4% and 10%, respectively (Figure 5B, C), based on the ratio of peak areas corrected for peak sensitivities. The Eu 4d spectra of the Eu CA-BNMG-1 beads revealed two sets of doublet peaks, with the dominant set (99%) corresponding to Eu­(III) states at binding energies of 136.3 eV (Eu 4d 5/2) and 141.7 eV (Eu 4d 3/2), while a minor peak (1%) at 122.5 eV indicated the presence of Eu­(II) (Figure 5C).^25,46^ Similarly, the Yb 4d spectra of Yb CA-BNMG-1 beads displayed four sets of peaks, with peaks (A) and (B) identified as satellite peaks of Yb­(III) (90%) and peak (C) attributed to Yb­(II) (10%) as shown in Figure 5B.^47,48^ The adsorption mechanism of Yb­(III) and Eu­(III) ions on CA-BNMG-1 composite beads proceeds through a dual coordination pathway involving nitrogen and oxygen donor atoms exclusively from the BNMG-1 MOF framework. Figure 5(D-I) presents high-resolution XPS spectra for the O 1s and N 1s regions, illustrating detailed chemical bonding states. In the pristine state, N 1s spectra exhibit a primary peak at 398.6 eV, which deconvolutes into two components corresponding to imine (398.4 eV) and amine (399.6 eV) nitrogen atoms within the imidazole-based ligands.^49^ These nitrogen sites, rich in lone electron pairs, act as primary coordination points for rare earth element binding. Upon adsorption of Yb­(III), the N 1s peak shifts markedly to 401.5 eV, indicating strong electron withdrawal due to coordination and the formation of protonated nitrogen species (−N^+^–Yb­(III)). An additional low-binding-energy peak at 396.8 eV suggests redistribution of electron density within the imidazole ring. The smaller ionic radius of Yb­(III) (0.868 Å) facilitates stronger orbital overlap with nitrogen, resulting in robust complexation. In contrast, Eu­(III) causes a more moderate N 1s shift to 400.29 eV, suggesting weaker interaction with nitrogen, consistent with its larger ionic radius (0.947 Å), which limits effective orbital overlap. The persistence of peaks near 398.6 and 399.14 eV after Eu­(III) adsorption indicates that a significant fraction of nitrogen sites remains uncoordinated.

**Figure 5:**
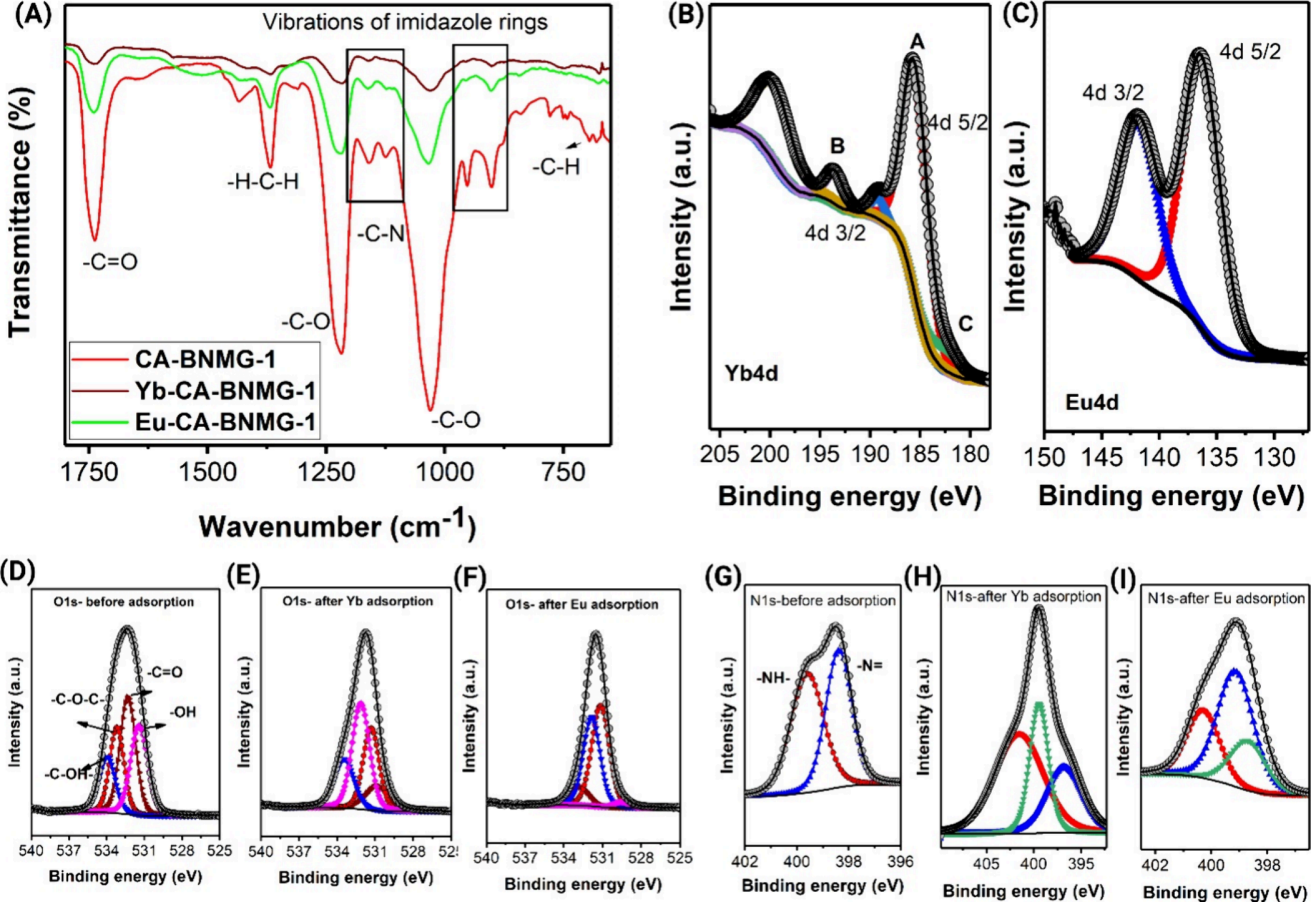
Post-adsorption analysis of the REE adsorbed composite beads. (A) FTIR spectra of composite beads before and after the Yb­(III) and Eu­(III) adsorption. (B–I) XPS high-resolution spectra of Yb­(III) and Eu­(III) adsorbed composite beads (B) Yb 4d (C) Eu 4d. (D–F) O 1s spectra before and after Yb and Eu adsorption. (G–I) N 1s spectra before and after Yb­(III) and Eu­(III) adsorption.

Simultaneously, O 1s XPS spectra exhibit shifts from 532.5 eV (pristine) to 531.7 and 531.6 eV for Yb­(III) and Eu­(III), respectively, confirming the involvement of oxygen atoms in metal coordination. These oxygen donors likely arise from multiple MOF framework components, bridging oxygens, and coordinated water molecules within pores. Notably, Eu­(III) shows a slightly larger O 1s shift than Yb­(III), indicating relatively stronger oxygen interaction, possibly due to its better spatial accommodation within the oxygen-rich sites. Together, the N 1s and O 1s spectral changes support a synergistic dual-site coordination mechanism in which REEs are partially dehydrated and simultaneously bind to both nitrogen and oxygen donors.^50,51^ Yb­(III) favors nitrogen-dominant coordination with secondary oxygen support, whereas Eu­(III) exhibits more balanced N/O interaction. This ionic radius-dependent coordination behavior provides a mechanistic rationale for the observed differences in adsorption.^52^

The results strongly indicate the formation of -N-REE and -O-REE bonds, which aligns with findings from the adsorption isotherm and kinetic studies, confirming that chemisorption governed by a monolayer adsorption mechanism is the predominant process driving adsorption. This adsorption mechanism was validated by recent articles and is called inner-sphere surface complexation.^24,33^ Overall, the post-adsorption analysis, encompassing SEM-EDS, FTIR, and adsorption isotherm and kinetics studies, conclusively demonstrates the strong affinity and effective interaction of Yb­(III) and Eu­(III) with the CA-BNMG-1 beads.

### 4.4 MOF Impregnation in CA Beads an Example of Safe and Sustainable by Design

The integration of BNMG-1 within the CA matrix embodies the SSbD framework (Table S4.1). This approach enhances the structural robustness and environmental safety of the adsorption process. By reducing copper ion leaching, the composite structure minimizes potential environmental Cu­(II) contamination. Moreover, the improved stability and efficiency in the separation of adsorbents after several reuses contributes to the sustainability of the CA-BNMG-1 material by facilitating recycling and reuse. Thus, this design strategy not only maintains the inherent affinity of BNMG-1 for REEs but increases its specificity for heavy REEs and aligns with broader goals of reducing waste and enhancing the lifecycle management of materials used in environmental cleanup processes.

To explore the environmental safety of the composite beads, the cytotoxicity of these beads were tested on ZF4 cells. The ZF4 cell line was established from 1-day-old zebrafish embryos and has recently gained interest as a potential early stage model to support the transition to *in vitro* methods as driven by the 3Rs directive (2010/63/EU) to replace, reduce and refine animal testing. The Trypan blue cytotoxicity assay was performed as it is less prone to artifacts than metabolic assays such as the MTT assay. Quantification of Cu­(II) in the cell culture supernatants using ICP-MS after 24h cell exposure clearly demonstrates that the impregnation of BNMG-1 into CA beads significantly reduced Cu­(II) release across all tested bead quantities. As shown in Figure 6A, the Cu release from CA-BNMG-1 beads was consistently lower than that of equivalent bare BNMG-1 MOFs, indicating the effectiveness of the CA matrix in regulating ion leaching. For a single bead, the CA-BNMG-1 beads released approximately 50 μg/mL of Cu­(II), compared to 100 μg/mL from the bare BNMG-1 MOFs. This trend continued at higher bead quantities, with the most pronounced difference observed at 10 beads, where CA-BNMG-1 beads released 500 μg/mL Cu­(II), while bare BNMG-1 MOFs released a substantially higher 2,500 μg/mL. The controlled Cu­(II) release from CA-BNMG-1 beads highlights the role of the CA matrix as a diffusion barrier, effectively limiting Cu­(II) leaching. For example, as shown in Figure S3.10, the release of Cu­(II) ions from CA-BNMG-1 beads in complex systems such as tap water (TW), artificial seawater (ASW) and canal water (CW) was less than 5% in 24 h, showcasing the stability of the prepared composite beads.

**Figure 6:**
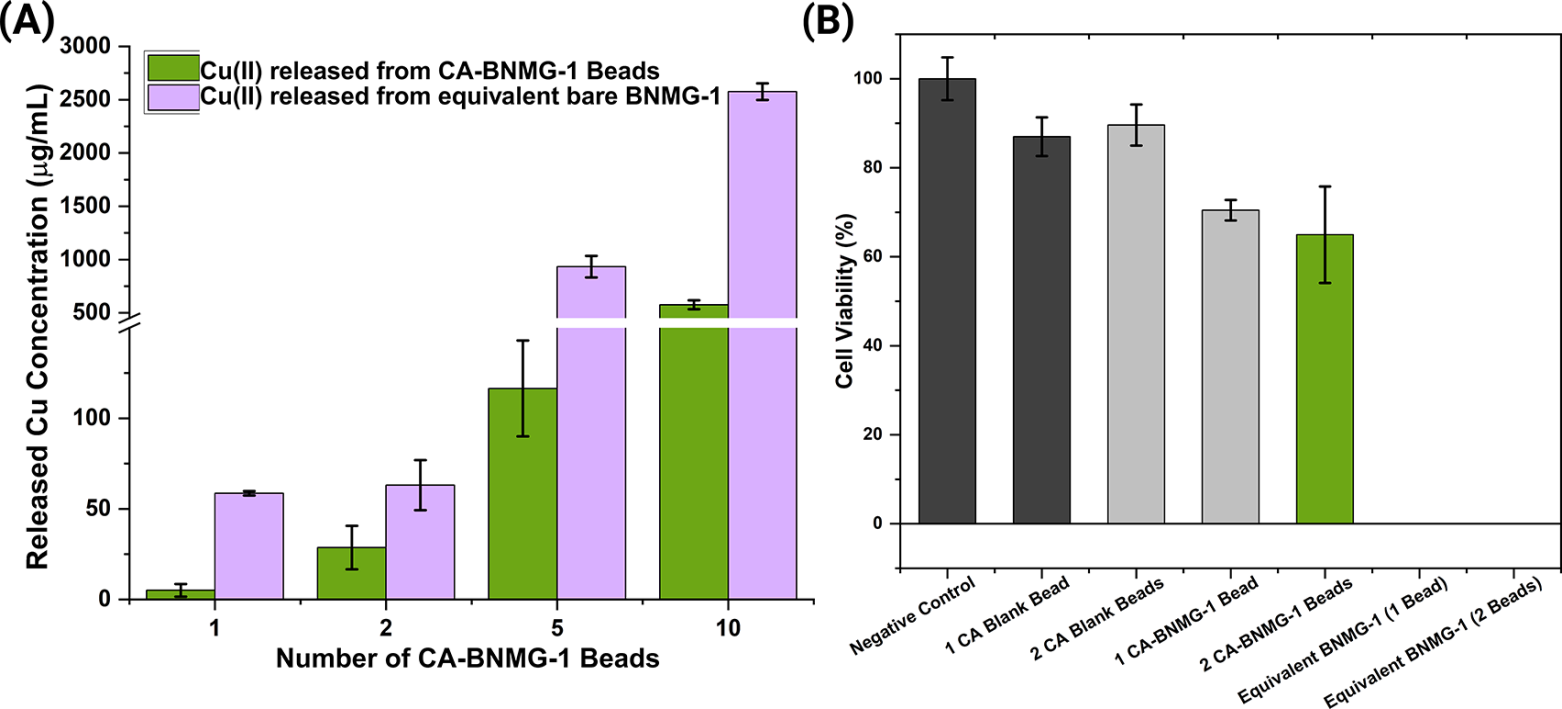
Safety assessment of CA-BNMG-1 beads. (A) Copper (Cu­(II)) concentration released from CA-BNMG-1 beads and equivalent bare BNMG-1 MOFs after 24 h of exposure in complete cell culture medium. The release was quantified using ICP-MS. The CA-BNMG-1 beads demonstrate a controlled release profile compared to the significantly higher Cu­(II) release observed from bare BNMG-1 MOFs across all tested quantities (1, 2, 5, and 10 beads). (B) Cell viability of ZF4 cells exposed to various conditions for 24 h, as assessed by the Trypan Blue exclusion assay. The error bars represent the standard deviation of two independent experiments conducted in triplicate (*n* = 3).

This is critical for reducing acute exposure risks, particularly in biological systems where excess Cu­(II) can lead to oxidative stress and cellular damage.^53^ Indeed, Cu­(II) released from nanoparticles is a well-documented driver of toxicity in biological systems, as highlighted by multiple studies.^54–57^ For example, the solubility of CuO nanoparticles directly correlates with their potential to act as copper-ion reservoirs, facilitating oxidative stress in intracellular environments.^55^ Furthermore, Cu­(II) ion release has been shown to enhance reactive oxygen species (ROS) production, disrupting cellular homeostasis and leading to damage to lipids, proteins, and DNA, as highlighted by recent findings.^56^ Previous work demonstrated that elevated levels of Cu­(II)­ions from nanoparticle dissolution resulted in significant toxicity, underscoring the importance of controlling ion release for safer nanomaterial design^57,58^ The cytotoxicity data in Figure 6B further corroborates the Cu­(II) release findings. ZF4 cells maintained high viability (above 70%) when exposed to 1 or 2 CA-BNMG-1 beads, demonstrating minimal cytotoxicity. In contrast, equivalent quantities of bare BNMG-1 MOFs result in no viable cells, even at the lowest BNMG-1 MOF concentration tested. These results emphasize the dual benefits of BNMG-1 impregnation into CA beads: the ability to regulate Cu­(II) release and the consequent reduction in cytotoxicity risks. The CA matrix not only provides a physical barrier to Cu­(II) leaching but also ensures a gradual release profile, making CA-BNMG-1 beads safer for ecosystems compared to their bare counterparts.

The economic feasibility of the CA-BNMG-1 composite beads was evaluated through unit economics analysis (detailed in Table S4.6, Supporting Information), revealing a laboratory-scale production cost of approximately £3.44 per gram. Table S4.1, Supporting Information further underscores this economic advantage by demonstrating how the beads align strongly with SSbD framework, particularly through significantly reduced operational complexity, minimal copper leaching, and improved recyclability. Compared to traditional REE recovery technologies such as solvent extraction and ion-exchange resins, which typically incur higher material costs, energy-intensive processes, and substantial secondary waste management expenses, the CA-BNMG-1 beads offer competitive economic benefits. Their simplified separation and regeneration steps further enhance their attractiveness by substantially lowering overall lifecycle costs. These attributes, supported by the quantitative analyses in Tables S4.1and 4.6, position CA-BNMG-1 beads as an economically attractive, environmentally responsible, and scalable solution, suitable for widespread adoption in critical-metal recovery applications.

## 5 Implications

This study successfully applies European Commission's SSbD framework to develop and characterize CA-BNMG-1 composite beads for REE recovery. By integrating BNMG-1 MOFs within a biobased CA matrix, the composite beads achieve exceptional adsorption efficiency and selectivity, even in complex multimetal systems. The CA encapsulation significantly reduces Cu­(II) ion leaching, mitigating toxicity risks and enhancing structural stability, key limitations of traditional MOFs. The beads exhibit superior separation factors for REEs such as Yb and Eu, and maintain 95% adsorption efficiency over multiple reuse cycles, ensuring long-term viability and cost-effectiveness. These properties establish prepared composite beads as a scalable and sustainable alternative for REE recovery, supporting resource circularity and minimizing environmental impacts.

The synthesis process adheres to green chemistry principles, using water-based MOF synthesis without hazardous solvents or extensive postprocessing. Embedding the MOF in a biodegradable, nontoxic CA matrix ensures safe and sustainable material design while optimizing resource efficiency and lowering production costs. A preliminary unit economics analysis (detailed in Supporting Information, Table S4.6) demonstrates the economic feasibility of CA-BNMG-1 beads, with a production cost of approximately £3.7 per gram at laboratory scale. These figures highlight the significant potential for cost reduction through scale-up, supporting practical industrial adoption. Future research will focus on scaling up the synthesis process while maintaining cost-effectiveness and structural integrity. Testing the beads in real-world waste streams, such as electronic waste leachates and mining effluents, will be crucial to assessing their resilience and adaptability. Long-term stability studies under diverse conditions will further validate their performance. Advanced spectroscopic and imaging techniques could provide deeper insights into the adsorption mechanisms, enabling further improvements in efficiency and selectivity. Addressing end-of-life management strategies will ensure full alignment with sustainable development goals. By combining the strong uptake capability of MOFs with the stability and safety of CA, these composite beads present a transformative solution for REE recovery, supporting a circular economy while minimizing ecological and human health risks.

## Supplementary Material



## Notes

The Supporting Information (SI) provides The Supporting Information is available free of charge at https://pubs.acs.org/doi/10.1021/acs.est.5c03112.

Additional details on the synthesis, characterization, and adsorption studies of CA-BNMG-1 composite beads; BNMG-1 MOF synthesis (S1.1), physicochemical characterization details (S1.2) using XRD, SEM-EDS, TEM, FTIR, TGA, XPS and ICP-MS, and batch adsorption/desorption experiments (S1.3) under controlled conditions (pH 5, temperature 25 °C); Adsorption kinetics, isotherms, and selectivity were assessed using simulated mine wastewater and e-waste leachates, analyzed via ICP-MS; Post-adsorption structural changes were examined using XPS, XRD, SEM-EDS, and FTIR; Cu­(II) leaching studies, safety assessment, and cost analysis (S4); Key results are summarized in Tables S4.1–S4.6 and Figures S3.1–3.10 (PDF)
