# Supplementary material for: Safe and Sustainable by Design MOF Beads for Selective Entrapment and Recovery of Rare Earth Elements
Source: Environ Sci Technol. 2025 Aug 1;59(31):16379–91. doi: 10.1021/acs.est.5c03112 (PMC12355941; doi:10.1021/acs.est.5c03112)
Supplement: Supplementary file 1 [file es5c03112_si_001.pdf]

Supporting Information for

Safe and Sustainable by Design MOFs beads for Selective Entrapment of Rare Earth Elements

Prathmesh Bhadane[ab], Pankti Dhumal[a], Emilie Brun[a], Andrew Britton[c], Iseult Lynch [a], Swaroop Chakraborty [a]*

[a]*School of Geography, Earth & Environmental Sciences, University of Birmingham, Edgbaston, B15 2TT, UK*

[b]*Materials Engineering, Indian Institute of Technology, Gandhinagar, India, 382355*

[c]School of Chemistry, University of Leeds, Leeds LS2 9JT, U.K.

*Corresponding author- s.chakraborty@bham.ac.uk

Summary:

- **Number of pages:** 22
- **Number of Figures:** 10 Figures (Figures S3.1 to S3.10)
- **Number of Tables:** 6 Tables (Tables S4.1 to S4.6)

S1. Supplementary Experimental Section:

S.1.1. Materials and methods

Materials: Cupric nitrate trihydrate ($Cu(NO_3)_2{\cdot}3H_2O$) [Merck], 2-Methyl Imidazole (2-MeIM) [Sigma-Aldrich], Cellulose acetate (Sigma-Aldrich), Dimethyl sulphoxide (DMSO), Metal salts-Ytterbium nitrate pentahydrate ($Yb(NO_3)_3.5H_2O$) (Sigma-Aldrich), Europium nitrate pentahydrate (Eu $(NO_3)_3.5\ H_2O$) (Sigma-Aldrich). Other REE/ metal salts, including metal nitrate and chloride salts, were of analytical chemistry grade and used as supplied.

S1.2. BNMG-1 MOF synthesis

Initially, 3.238 g of Cu $(NO_3)_2{\cdot}3H_2O$ was dissolved in 24 mL of ultrapure water, while 6.638 g of 2-methylimidazole (2-MeIM) was dissolved in 96 mL of ultrapure water. Both solutions were stirred separately for 30 minutes at room temperature. Subsequently, the solutions were combined and vigorously mixed using magnetic stirring. The resulting mixture was subjected to three washing steps with ultrapure water using centrifugation (7000 rpm, 10 minutes each) to remove any unreacted residues. The precipitate formed was vacuum-dried in an oven at 60°C for 12h. To ensure the removal of any residual aquatic solvent from the pores, the BNMG-1 MOF powder was further activated by heating in a vacuum oven at 110°C for 12 hours. The final BNMG-1 powder was then analyzed using various physicochemical characterization techniques, including Electron Microscopy and X-ray diffraction, to confirm its structural and compositional properties [1].

S1.3. Characterization of BNMG-1 MOF, CA and CA-BNMG-1 beads

X-ray diffraction (XRD) analysis was performed for phase identification using a Bruker D8-XRD instrument (Cu-Kα radiation) with a 2θ range of 5˚–50˚. The morphology and size of the prepared BNMG-1 nanosheets were examined using scanning electron microscopy (SEM, JEOL JSM7900F) and transmission electron microscopy (TEM, FEI, Themis 60-300). SEM was used to

analyze both the surface and cross-sectional morphology of the CA-BNMG-1 beads, while energy-dispersive X-ray spectroscopy (EDS) was employed to investigate the distribution of the MOF within the CA-BNMG-1 composite beads. Fourier transform infrared spectroscopy (FTIR) was conducted using a Perkin Elmer Spectrum Two FTIR instrument in the range of 400–4000 cm^{-1} to identify characteristic molecular bonds and functional groups associated with BNMG-1 and to confirm its presence within the CA beads. The thermal stability of BNMG-1, CA, and CA-BNMG-1 beads was assessed through thermogravimetric analysis (TGA) using a Perkin Elmer TGA 4000 instrument under a nitrogen atmosphere from 30˚C to 500˚C, with a heating rate of 10˚C per minute in an alumina crucible with initial mass of sample being approximately 5 mg. The copper (Cu (II)) content in BNMG-1 MOF and CA-BNMG-1 beads was quantified using inductively coupled plasma mass spectrometry (ICP-MS, Nexion 2000 ICP-MS, Perkin Elmer, USA) with Rhodium as internal standard following complete digestion of the samples in nitric acid (69%) under heating at 100°C.

S1.4. Batch adsorption experiments and post adsorption analysis

To evaluate the adsorption efficiency of BNMG-1 MOF, CA beads, and CA-MOF composite beads, a mixture of 10 REEs (20 mg/L each) was prepared at pH 5, covering both heavy REEs (Dy, Er, Tm, Yb, Lu, Tb) and light REEs (La, Nd, Sm, Eu). Adsorption tests were conducted by dispersing BNMG-1 MOF powder (1000 mg/L) directly into the REE solution, while CA and CA-BNMG-1 beads (5 beads/mL) were used separately. Samples were incubated for 24 hours in an orbital shaker, after which aliquots were collected. Aliquots collected after the adsorption experiments were diluted with 2% HNO_3 and subsequently analyzed for REEs and Cu(II) concentrations using inductively coupled plasma mass spectrometry (ICP-MS) to determine adsorption efficiency, distribution coefficients (Kd), separation factors (SF) and Cu (II) leaching from CA-BNMG-1 beads. ICP-MS measurements were carried out in Helium kinetic energy discrimination (KED) mode with a helium gas flow rate of 5 mL/min to minimise polyatomic

interferences and ensure accurate quantification of Cu(II) leaching and REE uptake. Unlike MOF powder, CA-based beads allowed easy separation without centrifugation.

For adsorption kinetics and isotherm studies, Yb and Eu were selected as representative REEs. Kinetic adsorption was examined using a 10 mg/L REE solution at pH 5, with 5 CA-BNMG-1 beads/mL, sampling over 0–48 hours under continuous stirring at temperature 25°C. Adsorption isotherms were determined with REE solutions ranging from 1–1000 mg/L. The initial pH of all adsorption experiments was adjusted to 5.0 ± 0.1 using 0.1 N HCl or 0.1 N NaOH. The kinetic data were fitted using pseudo-first-order and pseudo-second-order models, while linear and non-linear forms of Langmuir and Freundlich isotherm models assessed adsorption behaviour.

Reusability of CA-BNMG-1 beads was tested over five adsorption cycles (6 hours each) with 10 mg/L Yb and Eu solutions, followed by desorption using 1% HNO_3. After desorption with 1% HNO_3, the beads are intended for single-use disposal. This single desorption step was performed only after completing five consecutive adsorption cycles with fresh REE solutions (10 mg/L). The intent was to simulate practical operational conditions where the adsorbent is reused until near-saturation, followed by metal recovery. Post-desorption, the beads were not reused. Although the CA matrix remains structurally intact after exposure to 1% HNO_3, the MOF (BNMG-1) undergoes partial degradation under these acidic conditions, making the composite unsuitable for further reuse. Therefore, the desorption serves only to quantify and recover total REE uptake and not as a regeneration step. The selectivity of composite beads was tested using simulated mine wastewater and e-waste leachate, containing REEs alongside heavy metal ions (Mn, Cd, Ni, etc.) and coexisting ions (Na, Ca). Adsorption tests were conducted with 5 beads/mL for 24 hours, and post-adsorption metal concentrations were analysed using ICP-MS. Selectivity was assessed by calculating adsorption efficiency, distribution coefficients (K_d) for relative affinity comparison, and separation factors (SF) specific to the tested conditions. While K_d is traditionally applied to linear adsorption systems, it is used here as a simplified benchmarking metric to rank ion affinities under

identical experimental settings, consistent with precedents in complex matrices[2]. SF values reflect selectivity in the context of the simulated wastewater composition and operational parameters (5 beads/mL, 24 h contact time). Post-adsorption structural changes in BNMG-1 MOFs were examined using SEM-EDS, XPS, XRD, and FTIR before and after Yb(III) and Eu(III) adsorption. To demonstrate the mechanism of adsorption of Yb (III) and Eu (III) on CA-BNMG-1 beads, XPS spectra were measured in a SPECS enviroESCA NAP-XPS, using a Phoibos 150 NAP hemispherical analyzer with 1D delay line detectors. The x-ray source was a monochromated Al Kα anode (1486.7 eV) with a power of 42 W and an effective spot size on the sample of 0.3 mm allowing the individual beads to be measured. The beads were attached to an SEM stub with carbon tape and measured in an atmosphere of 10 mbar of nitrogen gas. This limits any potential differential charging on the surface. Survey spectra were taken for binding energies between 1100 and -3 eV at a pass energy of 100 eV, a step size of 1 eV, and a dwell time of 0.1 seconds on each step to identify any unexpected elements on the beads. Higher resolution scans were taken over specific elemental peaks at a pass energy of 50 eV, a step size of 0.1 eV, and a dwell time of 0.2 seconds. Binding energies were calibrated with the adventitious carbon peak set to 285 eV. Special care was taken for the Eu-containing beads due to some overlap between the C 1s peak and the Eu 4p ½ peak. Spectra were calibrated and analysed using CASAxps software [3].

S1.5. Safety assessment of the CA-BNMG-1 beads:

Zebrafish embryonic cell line (ZF4, ATCC, CRL-2050) was used to evaluate the cytotoxicity of CA-BNMG-1 beads using the Trypan Blue exclusion assay. ZF4 cells were cultivated in DMEM/F12 medium (31330038, Gibco), supplemented with 10% foetal bovine serum and 1% penicillin/streptomycin (15140122, Gibco) (complete cell culture media, CCM) in a humidified atmosphere of 5% CO_2 at 28°C. Cells were sub-cultured with 0.25% trypsin (15090046, Gibco, 5min at 28°C) after rinsing with phosphate buffered saline (PBS). Cells were seeded at a density of 80,000 cells per well in 500 µL of CCM in a 24-well plate and left to attach overnight. This

optimized seeding density ensured sub-confluency during the exposure period, allowing reliable evaluation of the effects of the test materials on cell viability. On the following day, ZF4 cells were exposed to different experimental conditions, including a negative control (CCM only), blank beads (1 and 2 blank CA beads per well), CA-BNMG-1 MOF-beads (1 and 2 beads per well), and equivalent MOF powder resuspended in CCM, i.e., 0.72 mg BNMG-1 (1 CA-BNMG-1 bead), and 1.44 mg BNMG-1 (2 CA-BNMG-1 beads) per well. Cells were exposed for 24 hours in total volume of 1 mL of CCM per well. Before exposure, beads were sterilized under UV light for 30 minutes to maintain aseptic conditions. Each condition was prepared in triplicate (n = 3) and two independent experiments were conducted.

Cytotoxicity was assessed using the Trypan Blue exclusion assay. Following exposure, beads and supernatants were collected separately. ZF4 cells were trypsinized and diluted in 0.4% Trypan Blue dye. The mixture was then examined using a hemocytometer to differentiate viable (unstained) from non-viable (stained) cells. The percentage of viable cells was calculated using the formula:

Viability (%) = (Number of viable cells in the sample / Number of viable cells in the negative control) *100

To quantify the release of Cu from the composite beads and equivalent BNMG-1 MOFs, the collected media were digested using an ashing mixture (3 : 1 molar ratio of HNO_3 and H_2O_2) of concentrated nitric acid (HNO_3) and 30% hydrogen peroxide (H_2O_2). This open digestion process was conducted in 20 mL glass vials under heating conditions at 120°C to ensure the complete breakdown of organic components and efficient extraction of metal ions into the solution. After digestion, the resulting samples were analyzed using ICP-MS (as described in Section 6.4) to determine the concentration of released Cu.

S2. Equations Used for adsorption studies

- Adsorption Efficiency $(\mathrm{RE})(\%) = \frac{C_0 - C_e}{C_0} \times 100$ Eq. S1
- Adsorption capacity $(q_e) = \frac{C_0 - C_e}{m} V$ Eq. S2
- Distribution Coefficient $(K_d) = \frac{C_0 - C_e}{C_e} \times \frac{V}{m}$ Eq. S3
- Separation factor (SF)= $\frac{K_d(metal-1)}{K_d(metal-2)}$ Eq. S4

Where, C_0 and C_e (mg/L) represents initial and equilibrium concentration of the REE ion in supernatant respectively. m (g) is adsorbent mass, V (mL) is solution volume, Kd (mL/g).

S2.1. Kinetics studies

<u>Pseudo first-order kinetics:</u>

$$\ln(Q_e - Q_t) = \ln Q_e - k_1 t \quad \text{Eq. S5}$$

<u>Pseudo second-order kinetics:</u>

$$\frac{t}{Q_t} = \frac{1}{k_2 Q_e^2} + \frac{t}{Q_e} \quad \text{Eq. S6}$$

Where, Q_e and Q_t represents the adsorption capacity at equilibrium and time t (mg/g), respectively. k_1 (min^{-1}) and k_2 (g $mmol^{-1}$ min^{-1}) represents parameters for kinetic rate constants for pseudo-first order and pseudo-second-order model, respectively.

S2.2. Adsorption isotherm studies

<u>Langmuir adsorption isotherm:</u>

$$Q_e = (Q_{max} K_L C_e)/(1+K_L C_e) \quad \text{Eq. S7}$$

<u>Freundlich adsorption isotherm:</u>

$$Q_e = K_f C_e^{1/n} \quad \text{Eq. S8}$$

$$\text{Separation factor } (R_L) = \frac{1}{1+K_L C_o} \qquad \text{Eq. S9}$$

Langmuir-Freundlich (Sips) isotherm model:

$$Q_e = Q_{max} (K^*C_e)^n / [1+(K^*C_e)^n] \qquad \text{Eq. S10}$$

Where, Q_{max} and Q_e represents the maximum (theoretical) and equilibrium adsorption capacity (mg/g) respectively, C_e is adsorbate concentration at equilibrium (mg/L), k_L (L/mg) and K_F (L/mg).

S3. Supplementary Results and Discussions

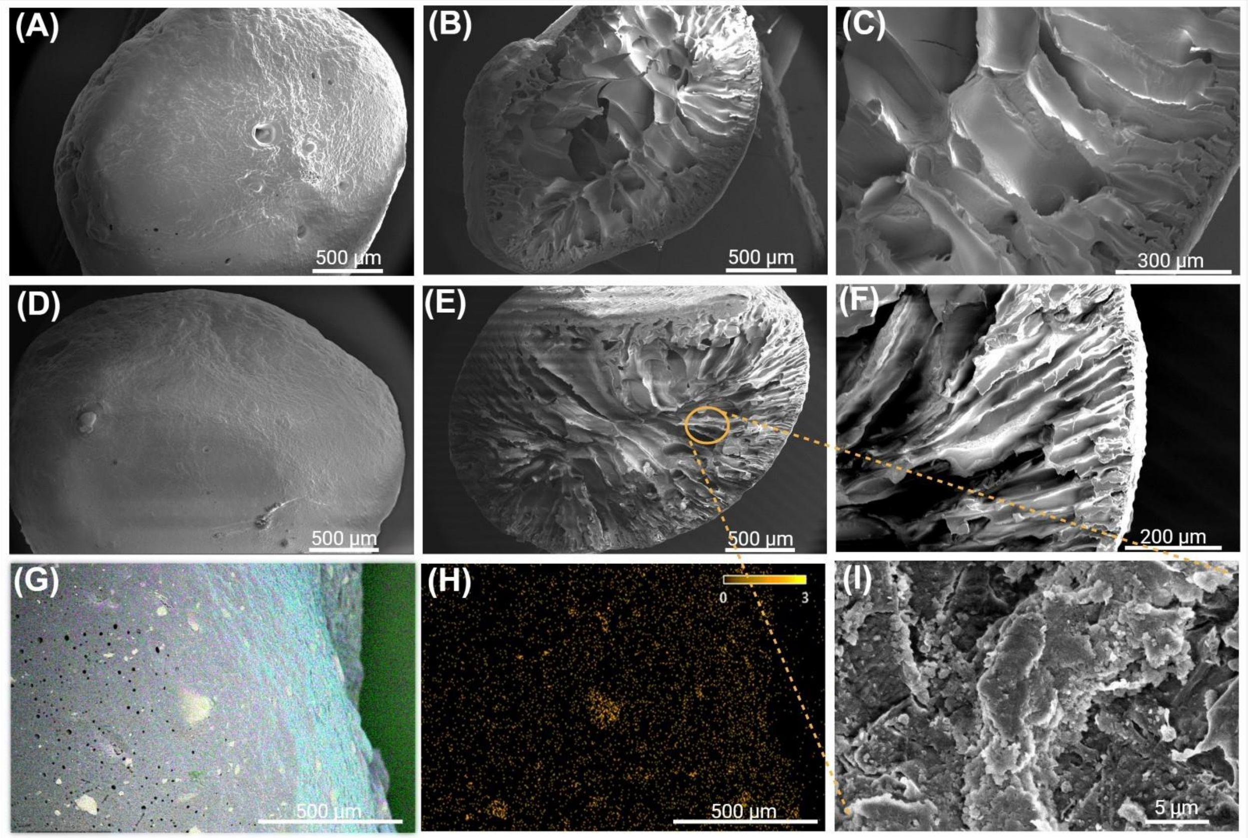

Figure S3.1: *SEM-EDS analysis of CA and BNMG-1 composite beads: (A–C) Surface morphology and cross-sectional micrographs of CA beads. (D–F, I) Surface morphology and cross-sectional micrographs of composite beads, highlighting the incorporation and distribution of MOF nanosheets within the CA matrix. (G, H) EDS mapping of CA-BNMG-1 beads confirming the elemental distribution of Cu from BNMG-1 in the composite structure.*

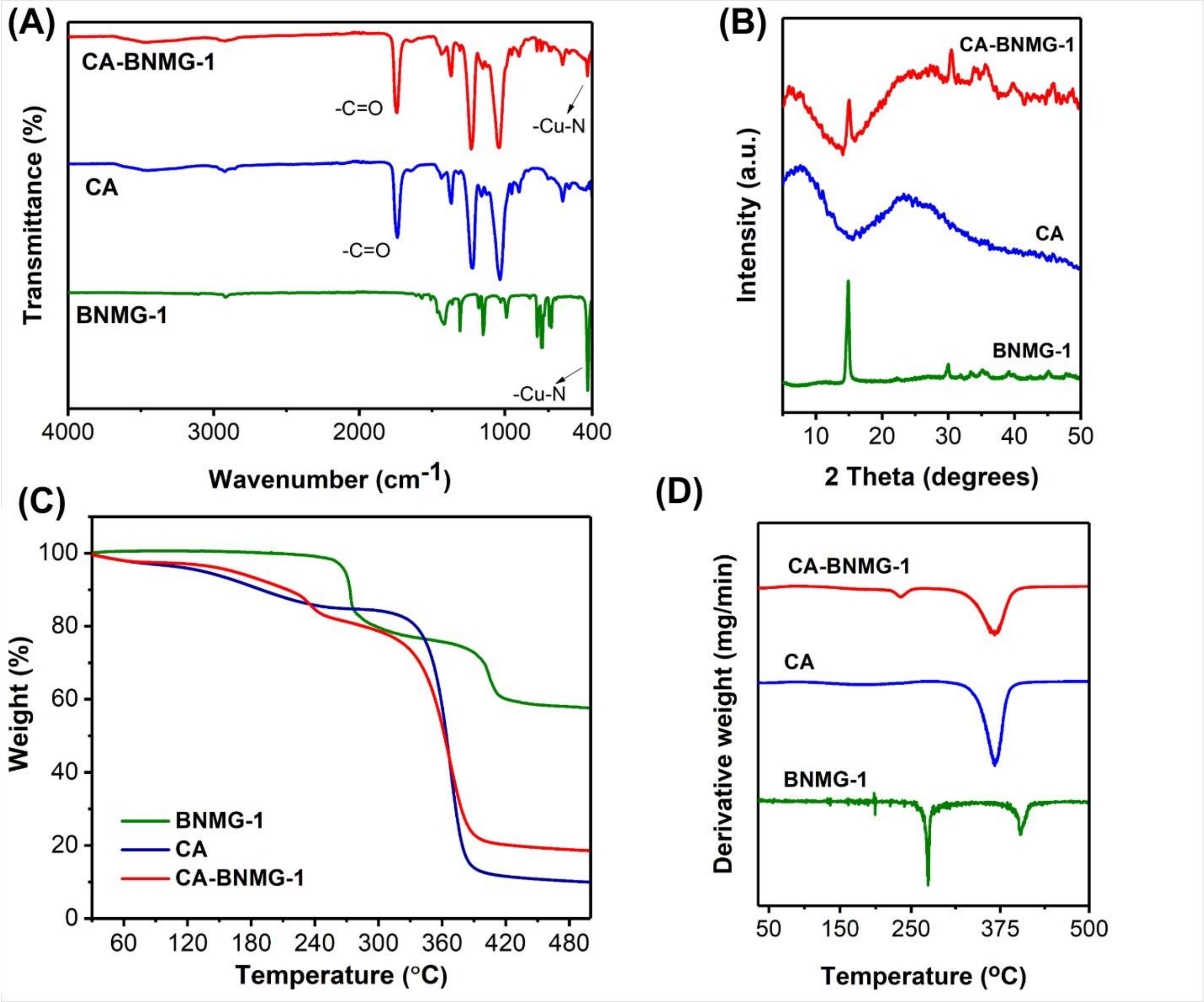

Figure S3.2: *Physicochemical characterization of BNMG-1 MOF and CA-MOF composite beads: (A) FTIR analysis confirming chemical stability and interactions of BNMG-1 within the CA matrix. (B) XRD patterns validating the structural stability of BNMG-1 MOF post-incorporation into CA beads. (C, D) TGA and DTG curves illustrating comparative thermal degradation behavior. The characterization data used in the figures were modified and reused from* [5]

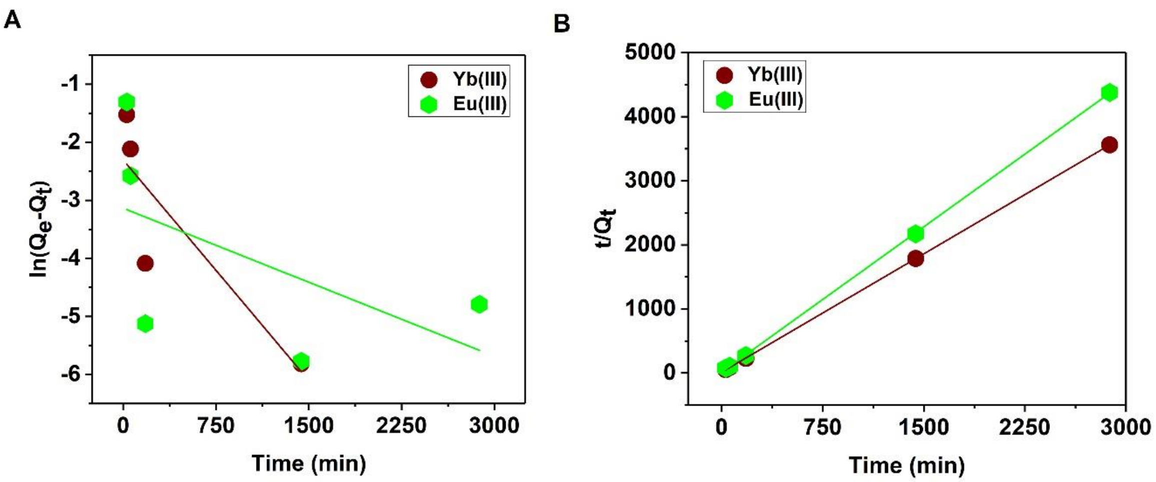

Figure S13.3: *Adsorption Kinetics- Pseudo first order and Pseudo second order model fitting*

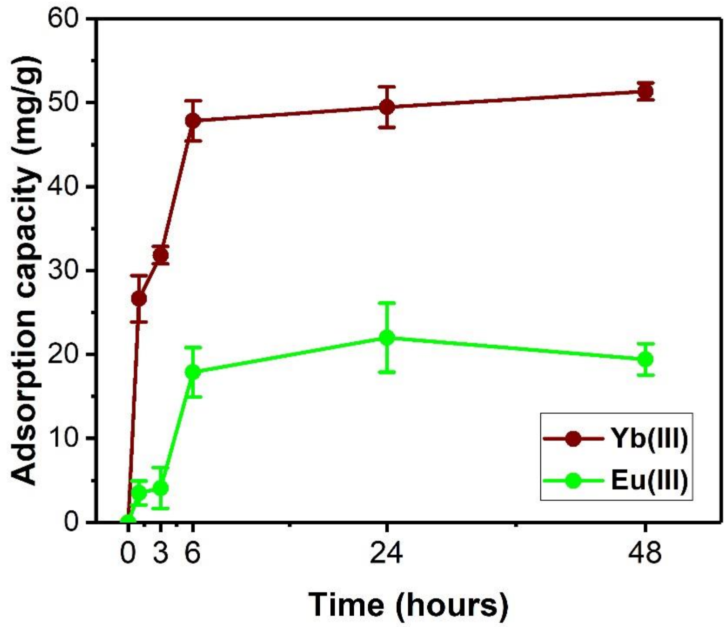

Figure S3.4. *Adsorption kinetics of Yb(III) and Eu(III) at an initial concentration of 1000 mg/L with 5 beads/mL. The adsorption capacity (mg/g) is plotted as a function of contact time (hours). Error bars represent standard deviations from triplicate experiments.*

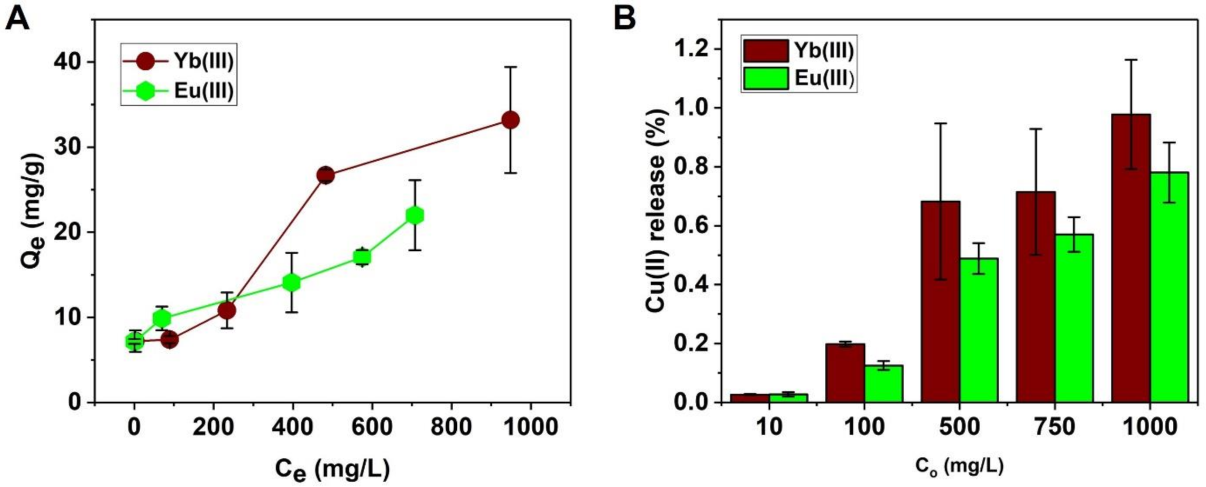

Figure S23.5: *(A) Adsorption capacity of Yb(III) and Eu(III) over CA-BNMG-1 beads with varying concentration (B)Cu(II) release with varying Yb(III) and Eu(III) concentrations.*

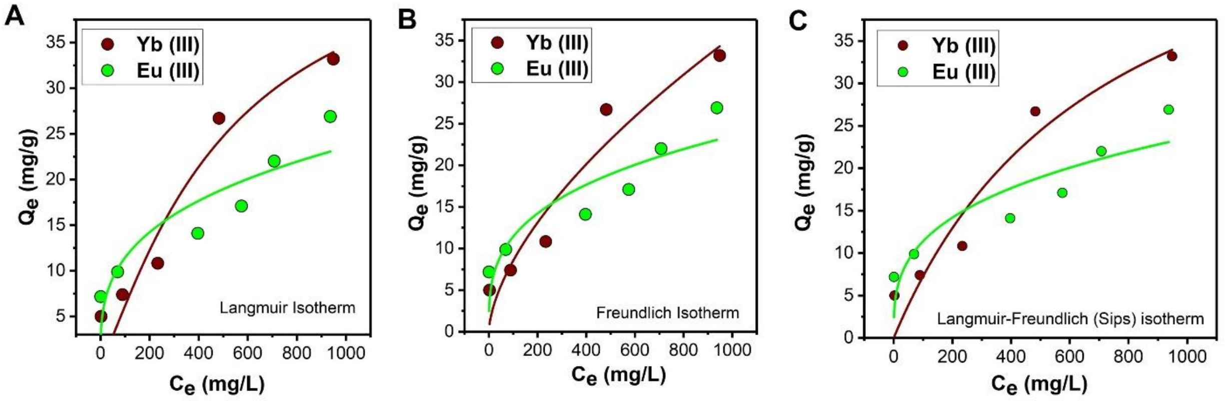

Figure S3.6: *Non-linear fitting of adsorption isotherm data of Yb(III) and Eu(III) using (A) Langmuir, (B) Freundlich (C) Langmuir-Freundlich (Sips) isotherm adsorption model fitting.*

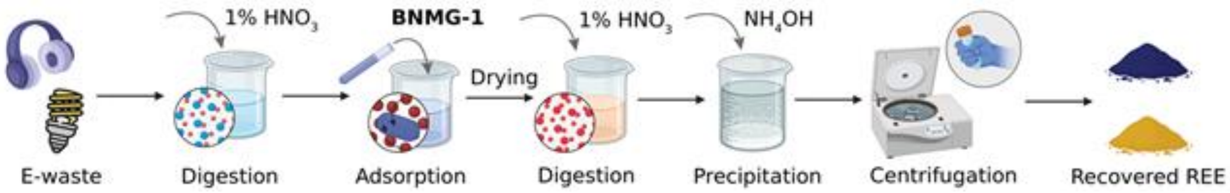

Figure S3.7 *REE recovery process reported in our previous work where BNMG-1 in powder form was used*[6]

S 3.7: After desorption: The 1000 mg/L Yb(III) treated CA-BNMG-1 beads were oven-dried and twenty beads underwent desorption in 5 mL of 1% HNO_3. From this process, 2 mL of eluent was collected, containing 337.4 mg/L Yb(III) and 234 mg/L Cu(II). Selective precipitation of $Yb(OH)_3$ was achieved by adding NH_4OH (1:1 v/v), which reduced dissolved Yb(III) to 51.2 mg/L. This resulted in an 84.5% recovery efficiency for Yb(III), while Cu(II) remained entirely soluble, confirming successful separation of the two metals.

S3.8- CA-BNMG-1 maintains selectivity for REEs over cations with different valencies

For this assessment, equal concentrations of each metal salt were combined to prepare a single mixed solution containing all ions at the same initial concentration. Batch adsorption experiments were conducted over 24 hours with continuous agitation at 200 rpm in an incubator shaker. After the adsorption period, aliquots were drawn, and residual concentrations of all ions were simultaneously determined. Experiment was performed in triplicate to ensure reproducibility, and standard deviations were calculated to assess data precision. The adsorption performance of CA-BNMG-1 demonstrated pronounced selectivity toward Yb(III), achieving a removal efficiency of 87.89%. In contrast, significantly lower adsorption efficiencies were observed for other metal ions present in the same mixed solution under identical conditions, with As(III), Cr(III), and Zr(IV) exhibiting removal rates of 29.43%, 29.58%, and 17.06%, respectively. While Zr(IV)'s lower uptake compared to Yb(III) highlights preferential binding to rare earth ions despite its higher charge density, the marked disparity between Yb(III) and similarly charged As/Cr (ionic radii: Yb(III) 0.985 Å vs. As(III) 0.58 Å, Cr(III) 0.615 Å) underscores selectivity governed by ionic radius and ligand-specific coordination rather than charge alone. The separation factor of Yb compared to As and Cr (3–5) confirms meaningful discrimination in trivalent-rich environments, validating CA-BNMG-1's applicability for REE recovery from complex matrices.

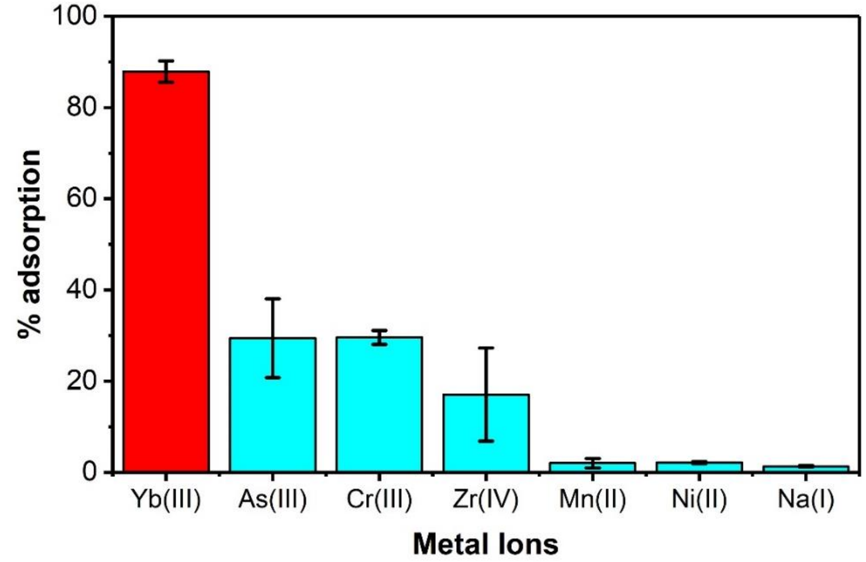

Figure S3.8: *Adsorption performance of Yb(III) in relation to metal ions with different valence states.*

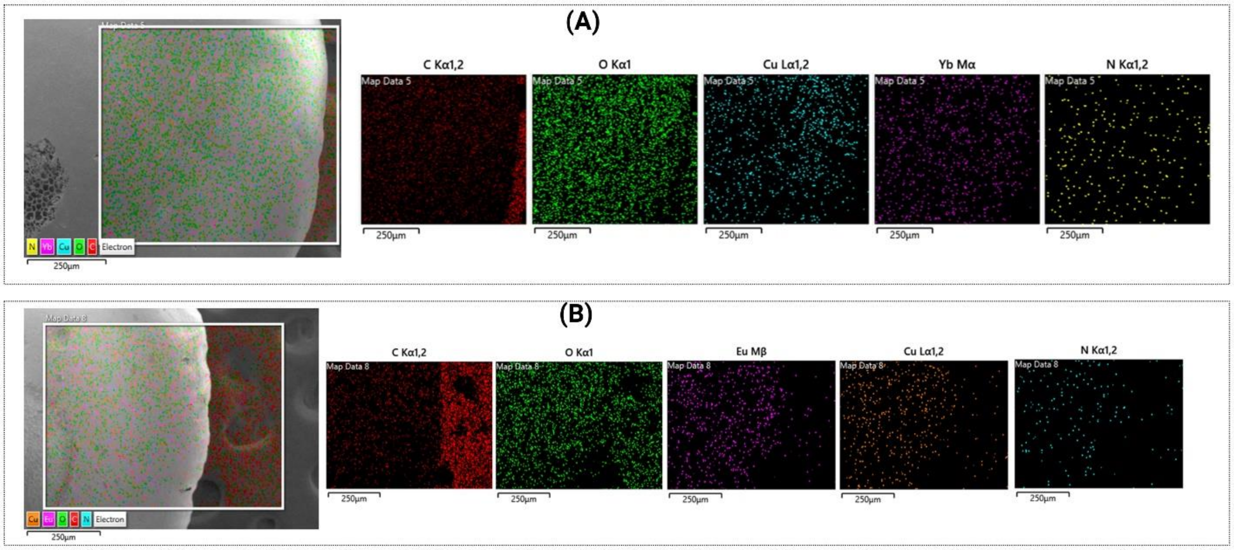

Figure S3.9: *SEM-EDS map confirming adsorption of (A) Yb(III) and (B) Eu(III) on the surface of composite beads*

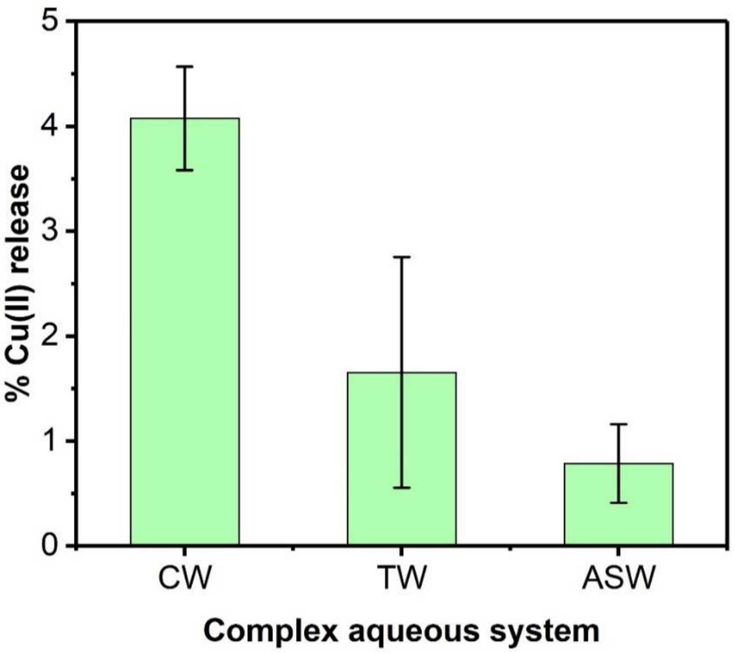

Figure S3.10: *Cu (II) release from CA-BNMG-1 beads in complex aqueous system, tap water (TW), canal water (CW) and artificial sea water (ASW)*

S4. Supporting information tables

Table S4.1: Safe and Sustainable by Design components of our MOFs Beads

Aspect / SSbD Step	How the CA-BNMG-1 beads fulfil the SSbD guidelines	Alignment with UN SDG Goals
**Green Chemistry**<br>(Step 1: Hazard assessment of chemicals and material.<br>Step 2: Human health and safety aspects in production / processing)	The BNMG-1-CA beads align with the *green chemistry principles* through a water-based synthesis method that eliminates the use of harmful organic solvents and ensures resource efficiency.<br>By incorporating a biodegradable cellulose acetate matrix, the composite *minimizes copper ion leaching*, enhancing safety and sustainability in rare earth element recovery.	• Responsible Consumption and Production (use of renewable & biodegradable resources)<br>• Climate action (reducing environmental impact of material synthesis)
**Economic Viability**<br>(Step 5: Social and Economic sustainability assessment)	The CA-BNMG-1 beads demonstrate economic viability with laboratory-scale preparation costs of 2.88 GBP for 400 mg of BNMG-1 (£7.20 / g of BNMG-1 MOFs) and 3.78 GBP for 1–1.2 g of CA-BNMG-1 beads (£3.44 / g of CA-BNMG-1 beads). This cost-effectiveness, coupled with high REE selectivity, highlights their potential for scalable and sustainable applications	• Decent Work and Economic Growth (cost-effective and scalable material production)<br>• Responsible Consumption and Production (use of renewable feedstocks)
**Ease of Separation**<br>(Step 4: Environmental Sustainability Assessment)	The millimeter-sized CA-BNMG-1 beads enable easy filtration using simple nets, eliminating the need for complex separation processes as required for fine powders. This significantly reduces operational complexity, enhancing scalability.	• Decent Work and Economic Growth (high potential for scale-up and use due to low infrastructure needs)<br>• Sustainable cities and Communities
**Metal Leaching / Instability**<br>(Step 3: Human Health and Environmental aspects in final application phase)	The formulation of the BNMG-1 MOFs into the CA beads reduces Cu^{2+} leaching by a factor of 20: <1% of Cu (II) releases from the beads in aqueous solution within 24h, compared to ~20% Cu (II) release from the bare BNMG-1 under identical conditions.	• Life on Land (reducing toxicity impact from mis-managed REE on ecosystems)<br>• Good Health and Well-being (via environmental remediation)
**Toxicity**<br>(Step 3: Human Health and Environmental aspects in final application phase)	Reduced Cu (II) exposure from CA-BNMG-1 beads significantly lowers toxicity risks, with Cu release capped at **100 µg/mL** for 2 beads compared to **500 µg/mL** from bare BNMG-1 MOFs. Consequently, cell viability remained above **80%** for CA-BNMG-1 beads, while it dropped below **50%** for bare BNMG-1 MOFs.	• Life on Land (reducing toxicity impact on ecosystems)<br>• Good Health and Well-being (via environmental remediation)

**Selectivity Towards REEs**<br>(Step 5: Social and Economic sustainability assessment)	The CA-BNMG-1 beads have high REE selectivity and reduced interference from other metals, making them effective for use in environmental remediation.	• REE recovery supports sustainable energy technologies<br>• Efficient resource recovery
**Recyclability & Reusability**<br>(Step 4: Environmental Sustainability Assessment)	95% adsorption efficiency retention after five cycles and improved water stability (<5% Cu release from in 96 h).	• Responsible Consumption and Production (Durable and sustainable materials)

Table S4.2: Comparison of CA-BNMG-1with other adsorbents in terms of Green Chemistry

Different adsorbents / Methods used for REE recovery	Outlook (Green Chemistry Perspective)	Examples
Polymeric	Requires post-functionalization with additional chemicals or other additives, often using synthetic reagents that may not be environmentally benign. Some polymers lack biodegradability, posing sustainability concerns.	• Quaternary ammonium functionalized cellulose<br>• sulfonated cellulose [7]
Silica based	Low adsorption capacity and poor stability in extreme pH conditions, leading to material degradation and potential environmental hazards. Silica synthesis methods often involve high-temperature processes that increase energy consumption.	• APTES functionalized silica[8]<br>• Amino-functionalized SBA-15[9]
Carbon based materials	Complex synthesis routes require toxic chemicals (e.g., strong acids, oxidizers) and high energy inputs (e.g., pyrolysis, chemical vapor deposition). Graphene oxide (GO) and reduced GO (rGO) primarily rely on oxygen functional groups, limiting selectivity and requiring additional functionalization.	• Biosynthesized reduced graphene oxide[10]<br>• pyrrolic-N doped activated carbon electrodes[11]
Other adsorbents (bio-char, zeolites etc.)	Limited adsorption capacity compared to advanced materials. Some byproduct-derived adsorbents (e.g., fly ash, coal ash) may contain residual toxic elements, affecting sustainability and environmental impact.	• [12]<br>• Bentonite[13]
Other MOFs	Many MOFs are synthesized using toxic organic solvents, hazardous metal precursors, and energy-intensive methods (e.g., solvothermal synthesis). Poor scalability and handling challenges (powder form) limit real-world applications.	• Zn-BTC[14]<br>• EDTA-chitosan modified ZIF-8[15]
Pyrometallurgy	High-temperature process (~1000–2000°C) leading to extremely high energy consumption and significant CO_2 emissions. Often requires fluxing agents and reducing agents that contribute to chemical waste and environmental pollution. Not aligned with green chemistry principles.	[16]
Hydrometallurgy	Uses strong acids and leaching agents (e.g., HCl, H_2SO_4, cyanide), leading to hazardous liquid waste generation. Though it operates at lower temperatures, the process involves chemical-intensive steps and often requires additional purification methods.	[16]
**This work- CA-BNMG-1**	Green, scalable, and sustainable synthesis of BNMG-1 MOF without toxic chemicals or extra energy input, fully	[6]

	aligned with green chemistry principles. The high adsorption capacity (>300 mg/g for REEs), combined with cellulose acetate (biopolymer) integration, addresses key issues like toxicity, energy efficiency, and environmental impact, making it a viable and sustainable solution for industrial-scale applications.	

*T**able S4.3**: FTIR spectral assignments of functional groups in BNMG-1, cellulose acetate (CA), and CA-BNMG-1 composites.*

Functional groups	BNMG-1	CA	CA-BNMG-1
-Cu-N-	400–500 cm^{-1}	Absent	Present
Ester C=O	Absent	1741 cm^{-1}	1749 cm^{-1} (shifted)
Hydroxyl (-OH)	Weak	Strong	Reduced

***Table S4.4:** Adsorption Kinetics and adsorption isotherm parameters for Yb (III) and Eu (III) adsorption using CA-BNMG-1 beads (Linear fitting).*

	**Pseudo-first order**			**Pseudo-second order**		
	**K_1 (min)**	**Q_e (mg/g)**	**R^2**	**K_2 {g/ (mg* min)}**	**Q_e (mg/g)**	**R^2**
**Yb (III)**	0.00253	0.101	0.7714	0.1376	0.811	0.9999
**Eu (III)**	$8.50*10^{-4}$	0.0435	0.3132	0.2059	0.661	0.9998
	**Langmuir Model**			**Freundlich Model**		
	**Q_{max} (mg/g)**	**B (L/mg)**	**R^2**	**K_f (L/mg)**	**n**	**R^2**
**Yb (III)**	42.48	0.0029	0.7063	4.245	4.05	0.6199
**Eu (III)**	24.87	0.00482	0.7983	5.405	6.61	0.3723

Table S4.5: Effect of anions on the adsorption of REEs onto different adsorbents

MOF	Functional Groups	Anion Impact	Reference
NCU-1	Carboxyl, triazole	Anions minimally affect adsorption due to size-selective nanotraps.	[17]

MIL-101-PMIDA	Phosphonic, carboxyl	Competing anions (e.g., PO_4^{3-}) reduce Gd^{3+} uptake by 15–20% at pH 3.	18
RE-DOBDC	Fluorinated clusters	SO_4^{2-} and NO_3^- have negligible impact on REE adsorption due to cluster stability.	19

Table S4.6 Unit Economics of BNMG-1 and CA-BNMG-1 beads (Ref: sigmaaldrich.com)

Component	BNMG-1 MOFs	CA-BNMG-1 Beads
**Raw Materials**		
Copper Nitrate [$Cu(NO_3)_2 \cdot 3H_2O$]	3.238 g (£0.453)	—
2-Methylimidazole (2-MeIM)	6.638 g (£2.23)	—
Cellulose Acetate (CA)	—	1 g (£0.178)
Dimethyl Sulfoxide (DMSO)	—	10 mL (£2.06)
BNMG-1 Content	—	200 mg (£1.437)
**Utilities**		
Ultrapure Water	120 mL (negligible cost)	500 mL (negligible cost)
Vacuum Oven Drying (Energy)	12 h at 110°C (£0.192)	8 h at 60°C (£0.12)
**Total Cost per Batch**	£2.88	£3.78
**Yield**	400 mg	1.1 g (1 g CA + 200 mg BNMG-1)
**Cost per Gram**	£7.20/g	£3.44/g

This estimate is based on laboratory-scale preparation. In large-scale production, the costs of materials would decrease significantly due to economies of scale and automation.

Reference-

(1) Goyal, P.; Menon, D.; Jain, P.; Prakash, P.; Misra, S. K. Linker Mediated Enhancement in Reusability and Regulation of Pb(II) Removal Mechanism of Cu-Centered MOFs. *Sep Purif Technol* **2023**, *318*, 123941. https://doi.org/10.1016/J.SEPPUR.2023.123941.

(2) Wu, R.; Hong, B.; Xue, C.; Chen, Z.; Chen, Z. ZIF-8 Used for the Selective Recovery of Heavy Rare Earth Elements from Mining Wastewater. *Environ Sci Technol* **2024**, *58* (22), 9612–9623. https://doi.org/10.1021/ACS.EST.3C10653/ASSET/IMAGES/LARGE/ES3C10653_0006.JPEG.

(3) Cho, E.; Oh, S. Surface Valence Transition in Trivalent Eu Insulating Compounds Observed by Photoelectron Spectroscopy. *Phys Rev B* **1999**, *59* (24), R15613–R15616. https://doi.org/10.1103/PHYSREVB.59.R15613.

(4) Zhang, C.; Chen, W.; Owens, G.; Chen, Z. Recovery of Rare Earth Elements from Mine Wastewater Using Alginate Microspheres Encapsulated with Zeolitic Imidazolate Framework-8. *J Hazard Mater* **2024**, *471*, 134435. https://doi.org/10.1016/J.JHAZMAT.2024.134435.

(5) Bhadane, P.; Chakraborty, S. Cellulose Acetate-NanoMOF Beads: A Safe, Sustainable and Scalable Solution for Pb Remediation in Complex Water Systems. *Environ Sci Nano* **2025**. https://doi.org/10.1039/D5EN00056D.

(6) Bhadane, P.; Menon, D.; Goyal, P.; Reza Alizadeh Kiapi, M.; Kanta Satpathy, B.; Lanza, A.; Mikulska, I.; Scatena, R.; Michalik, S.; Mahato, P.; Asgari, M.; Chen, X.; Chakraborty, S.; Mishra, A.; Lynch, I.; Fairen-Jimenez, D.; Misra, S. K. A Two-Dimensional Metal-Organic Framework for Efficient Recovery of Heavy and Light Rare Earth Elements from Electronic Wastes. *Sep Purif Technol* **2025**, *360*, 130946. https://doi.org/10.1016/J.SEPPUR.2024.130946.

(7) Bekchanov, D.; Mukhamediev, M.; Yarmanov, S.; Lieberzeit, P.; Mujahid, A. Functionalizing Natural Polymers to Develop Green Adsorbents for Wastewater Treatment Applications. *Carbohydr Polym* **2024**, *323*, 121397. https://doi.org/10.1016/J.CARBPOL.2023.121397.

(8) Aguado, J.; Arsuaga, J. M.; Arencibia, A.; Lindo, M.; Gascón, V. Aqueous Heavy Metals Removal by Adsorption on Amine-Functionalized Mesoporous Silica. *J Hazard Mater* **2009**, *163* (1), 213–221. https://doi.org/10.1016/J.JHAZMAT.2008.06.080.

(9) Ramasamy, D. L.; Wojtuś, A.; Repo, E.; Kalliola, S.; Srivastava, V.; Sillanpää, M. Ligand Immobilized Novel Hybrid Adsorbents for Rare Earth Elements (REE) Removal from Waste Water: Assessing the Feasibility of Using APTES Functionalized Silica in the Hybridization Process with Chitosan. *Chemical Engineering Journal* **2017**, *330*, 1370–1379. https://doi.org/10.1016/J.CEJ.2017.08.098.

(10) Yang, Y.; Weng, X.; Chen, Z. Recovery of Rare Earth Elements from Mine Wastewater Using Biosynthesized Reduced Graphene Oxide. *J Colloid Interface Sci* **2023**, *638*, 449–460. https://doi.org/10.1016/J.JCIS.2023.02.004.

(11) Zhao, F.; Chen, S.; Xiang, H.; Gao, T.; Wang, D.; Wei, D.; Sillanpää, M.; Ke, Y.; Tang, C. J. Selectively Capacitive Recovery of Rare Earth Elements from Aqueous Solution onto Lewis Base Sites of Pyrrolic-N Doped Activated Carbon Electrodes. *Carbon N Y* **2022**, *197*, 282–291. https://doi.org/10.1016/J.CARBON.2022.06.033.

(12) Pinto, J.; Colónia, J.; Abdolvaseei, A.; Vale, C.; Henriques, B.; Pereira, E. Algal Sorbents and Prospects for Their Application in the Sustainable Recovery of Rare Earth Elements from E-Waste. *Environ Sci Pollut Res Int* **2023**, *30* (30), 74521. https://doi.org/10.1007/S11356-023-27767-8.

(13) Mosai, A. K.; Chimuka, L.; Cukrowska, E. M.; Kotzé, I. A.; Tutu, H. The Recovery of Rare Earth Elements (REEs) from Aqueous Solutions Using Natural Zeolite and Bentonite. *Water Air Soil Pollut* **2019**, *230* (8), 1–17. https://doi.org/10.1007/S11270-019-4236-4/METRICS.

(14) Wu, J.; Li, Z.; Tan, H.; Du, S.; Liu, T.; Yuan, Y.; Liu, X.; Qiu, H. Highly Selective Separation of Rare Earth Elements by Zn-BTC Metal-Organic Framework/Nanoporous Graphene via In Situ Green Synthesis. *Anal Chem* **2021**, *93* (3), 1732–1739. https://doi.org/10.1021/ACS.ANALCHEM.0C04407.

(15) Feng, S.; Du, X.; Bat-amgalan, M.; Zhang, H.; Miyamoto, N.; Kano, N. Adsorption of REEs from Aqueous Solution by EDTA-Chitosan Modified with Zeolite Imidazole Framework (ZIF-8). *International Journal of Molecular Sciences 2021, Vol. 22, Page 3447* **2021**, *22* (7), 3447. https://doi.org/10.3390/IJMS22073447.

(16) Gkika, D. A.; Chalaris, M.; Kyzas, G. Z. Review of Methods for Obtaining Rare Earth Elements from Recycling and Their Impact on the Environment and Human Health. *Processes 2024, Vol. 12, Page 1235* **2024**, *12* (6), 1235. https://doi.org/10.3390/PR12061235.

(17) Ilgen, A. G.; Sikma, R. E.; Sava Gallis, D. F.; Leung, K.; Sun, C.; Song, B.; Sanchez, K. M. M.; Smith, J. G. Local Coordination Environment of Lanthanides Adsorbed onto Cr- and Zr-Based Metal-Organic Frameworks. *ACS Appl Mater Interfaces* **2024**, *16* (36), 48536–48546. https://doi.org/10.1021/ACSAMI.4C09445/ASSET/IMAGES/LARGE/AM4C09445_0007.JPEG.

(18) Hu, Q. H.; Song, A. M.; Gao, X.; Shi, Y. Z.; Jiang, W.; Liang, R. P.; Qiu, J. D. Rationally Designed Nanotrap Structures for Efficient Separation of Rare Earth Elements over a Single Step. *Nature Communications 2024 15:1* **2024**, *15* (1), 1–7. https://doi.org/10.1038/s41467-024-45810-1.

(19) Christian, M. S.; Fritzsching, K. J.; Harvey, J. A.; Sava Gallis, D. F.; Nenoff, T. M.; Rimsza, J. M. Dramatic Enhancement of Rare-Earth Metal-Organic Framework Stability Via Metal Cluster Fluorination. *JACS Au* **2022**, *2* (8), 1889–1898. https://doi.org/10.1021/JACSAU.2C00259/SUPPL_FILE/AU2C00259_SI_002.ZIP.
